# Emergence of chiral multi-armed spirals in an open system of migrating cells under continuous cell supply

**DOI:** 10.1038/s41467-026-78179-4

**Published:** 2026-10-26

**Authors:** Masayuki Hayakawa, Biplab Bhattacherjee, Hidekazu Kuwayama, Tatsuo Shibata

**Affiliations:** 1 Laboratory for Physical Biology, RIKEN Center for Biosystems Dynamics Research, Kobe, Japan; 2 Department of Mechanical Engineering, Kyoto Institute of Technology, Kyoto, Japan; 3 Faculty of Life and Environmental Sciences, University of Tsukuba; Tennodai, 1-1-1, Tsukuba, Ibaraki, Japan

**Keywords:** Statistical physics, thermodynamics and nonlinear dynamics, Biological physics

## Abstract

From molecular assemblies to multicellular tissues, chirality organizes living and active-matter systems into striking collective states. Across cellular collectives and active materials, weak microscopic chiral cues in motion and interaction can systematically skew how constituents coordinate, flow, and break spatial symmetries. Yet the principles that govern chiral ordering in open systems, where elements are continuously supplied and removed, remain poorly understood. Here, we show that chemotaxis-deficient *Dictyostelium discoideum* mutants (KI cells) form centimeter-scale, clockwise, multi-armed spirals. Each arm is a traveling band produced by short-range alignment interactions and guided by a polar-ordered rotating core that encircles the cell source. A subtle clockwise bias in single-cell migration is amplified by collective ordering into tissue-scale chirality. To uncover the minimal ingredients, we developed an open chiral Vicsek model and found that intrinsic chirality, polar alignment, and continuous cell supply are the key factors. Our study establishes a general route by which weak microscopic chirality and sustained material flux generate macroscopic chiral order, offering new insight into chiral patterning in multicellular systems.

## Abstract (web summary)

Living and active-matter systems can develop large-scale chiral patterns from weak microscopic biases, but how such chirality emerges in open, continuously supplied systems remains poorly understood. Here, the authors combine experiments in *Dictyostelium discoideum* with an open chiral Vicsek model to show that intrinsic cell chirality, polar alignment, and continuous cell supply are sufficient to amplify a subtle migration bias into centimeter-scale rotating spiral patterns.

## Introduction

Chirality plays a fundamental role in shaping emergent properties across diverse physical, biological and material systems. Active systems, composed of self-propelled elements that continuously consume energy to sustain motion, exhibit especially rich behaviors when chirality is present^1,2^. Chiral active particles, spanning biological molecular motors^3^ and microswimmers^4–6^ to synthetic colloidal rotors^7^, display a variety of intriguing emergent macroscopic behaviors, including chiral edge currents^5,7–10^, odd diffusivity^11,12^, vortex lattices^3,4^, enhanced flocking^13,14^, phase separation into coherently rotating droplets^13,14^ and even condensation into synchronized vortices and rotating polar packets^15^.

Biological systems are constructed from chiral building blocks such as proteins and DNA. The collective behaviors of these chiral molecular processes^16,17^ manifest as active chiral properties in cells^18–25^, a phenomenon known as cell chirality^26,27^. Although cell chirality is considered to underlie left-right asymmetry in organs and bodies^27–31^, the mechanisms that transmit chiral information from the cellular level to the tissue and organ scales remain largely unknown. Insight into this multiscale transfer may come from the self-organization of chiral active matter^1^.

Most studies of chiral active matter have examined closed systems in which the number of constituent elements is conserved. During morphogenesis, however, tissues are often open systems in which cell numbers change through proliferation and cell influx from a source. Neural crest cells, for example, delaminate from specific regions of the dorsal neural tube, thereby providing a localized source of migratory cells, and migrate extensively throughout the embryo. In such open systems, which continuously exchange matter with their surroundings, tissue-level structures can arise and persist under a steady influx of new elements. However, whether and how weak microscopic chirality of the constituents, when coupled to sustained material flux in open systems, can generate distinct macroscopic chiral order remains unclear.

Here, by combining experiments on a chemotaxis-deficient mutant of *Dictyostelium discoideum* with a minimal open chiral Vicsek model, we show that weak single-cell chirality, polar interactions, and continuous cell supply generate a centimeter-scale pattern of clockwise-winding multi-armed spirals. These spirals are organized and sustained by a polar-ordered rotating core that encircles the cell source. Together, these results establish a general route by which weak microscopic chirality is amplified into macroscopic chiral order in open systems.

## Results

### Self-organization of a multi-armed spiral structure in mutant *Dictyostelium* cells

**Fig. 1:**
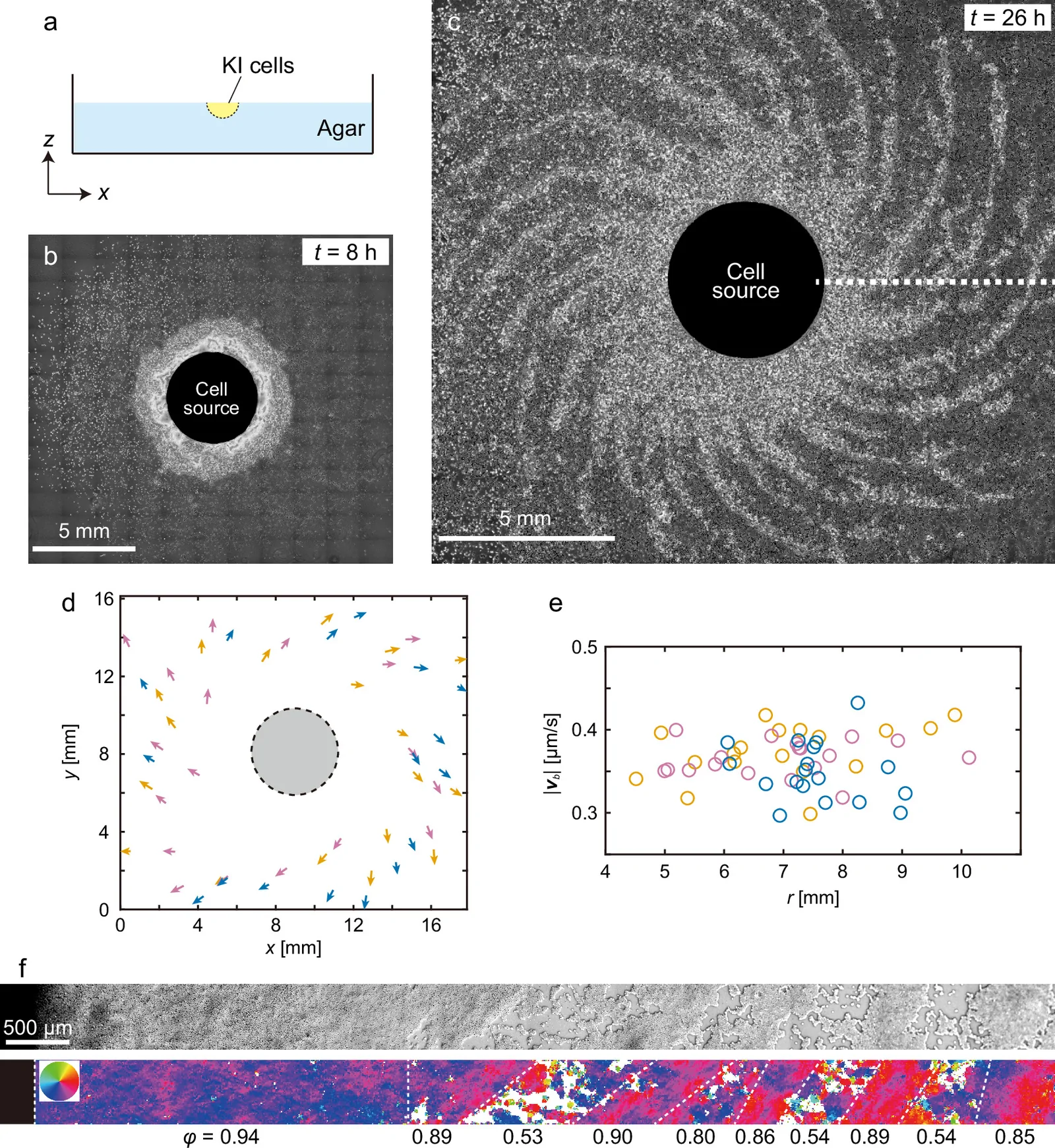
Large-scale chiral pattern formation of KI cells. **a** Schematics of experimental setup. **b** Formation of an annular structure around the cell source at 8 h. The cell density is high in the brighter region. **c** Multi-armed spiral pattern formation with the rotating core around the cell source at 26 h. See also Supplementary Movie 1. *N* = 3 independent experiments were repeated with similar results. **d** Spatial distribution of velocity of traveling bands. The velocity was measured as the displacement of each band along its local normal per unit time in the three independent experiments shown as different colors. *N* = 3 independent experiments. **e** Dependence of speed of the traveling bands on the distance (r) from the center. **f** High-magnification DIC image and particle image velocimetry (PIV) analysis at $t=26\,{{\rm{h}}}$ along the narrow window shown as a dotted line in (**c**). The colors indicate the direction of motion obtained by PIV, as shown in the color wheel. The numbers indicate the global polar order parameter ϕ calculated for the regions separated by the dotted lines. Source data are provided as a Source Data file.

To examine how activity and chirality interact in an open system, we employed a mutant strain of *Dictyostelium discoideum* known as the KI strain. KI cells lack chemotactic ability and therefore fail to aggregate even under starvation^32^. Instead, they display a distinctive collective behavior: a segregation of cell density that propagates as traveling bands^33,34^. Although a chiral bias in KI cells has not been established, wild-type *Dictyostelium* cells exhibit a slight clockwise migration bias^35^. Therefore, the KI strain offers an ideal model for probing the behavior of active chiral matter in an open system. To investigate this behavior under continuous cell supply, we designed an assay in which cells emerged gradually from defined locations. A pellet of starved KI cells was placed in a 2 mm–radius dimple on the surface of a non-nutrient agar gel (Fig. 1a). Within a few hours the cells began migrating outward, turning the pellet into a persistent cell source. The outward-moving population first formed a dense annulus around the source (Fig. 1b, Supplementary Movie 1). As time progressed, cells dispersed beyond this annulus, and multiple traveling bands arranged periodically around the circumference produced a centimeter-scale, multi-armed spiral that rotated clockwise, revealing large-scale chiral pattern formation (Fig. 1c and Supplementary Movie 1).

Velocity analysis revealed that the traveling bands propagated radially outward from the center while rotating clockwise (Fig. 1d). The propagation speed of the bands, V, was uniform across positions (Fig. 1e), with a mean speed of $0.363\,{{\rm{\mu }}}{{\rm{m}}}/{{\rm{s}}}$.

The pattern consists of a dense annular region around the cell source, surrounded by a traveling band region. To examine how cell motion differs across these regions, we performed particle image velocimetry (PIV) analysis on high-magnification differential-interference-contrast (DIC) images acquired in a rectangular window extending radially from the center to the periphery (Fig. 1f top). The color in Fig. 1f bottom indicates the direction of cell motion. Immediately adjacent to the dark cell source region on the far left is the dense annulus, where velocity is predominantly oriented downward (dark blue and magenta colors). Thus, the cells in the dense annular region rotate clockwise around the cell source. Directional alignment was quantified by the global polar order parameter $\phi=\left|\left\langle v/\left|v\right|\right\rangle \right|$, where v is the local PIV velocity. The high order parameter (*ϕ* = 0.94) confirms that this region is polar-ordered. Because it rotates clockwise around the field center, we refer to it as the rotating core. The rotating core was already present before the multi-armed spiral appeared (Fig. 1b) and displayed a similar degree of order (Supplementary Fig. 1a, b). In the traveling band region the order parameter remained high (ϕ=0.89, 0.90, 0.86, 0.89 and 0.85 from the left), whereas most gaps, except one region, between bands showed markedly lower alignment (ϕ=0.53, 0.80, 0.54, 0.54). These data indicate that in the region around the rotating core KI cells self-organize into polar-ordered, high-density traveling bands that propagate through a low-density, disordered gas of randomly migrating cells (Fig. 1f).

**Fig. 2:**
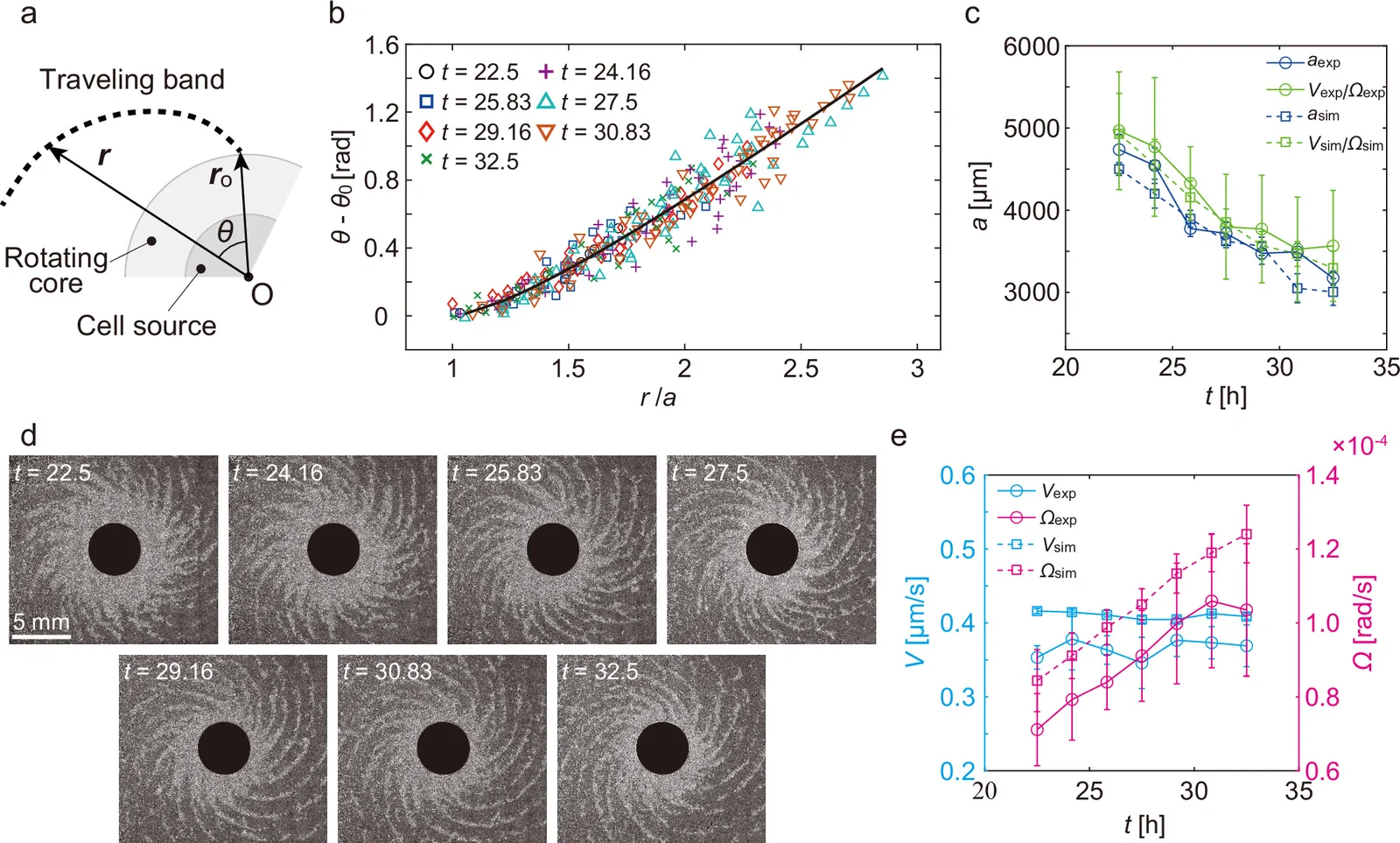
Shape of spiral pattern follows an involute of a circle. **a** Polar coordinates for the shape measurement of the traveling bands. **b** Shape analysis of the traveling band. The shape of the traveling band is well described by an involute of a circle given in Eq. (1). For each point along the traveling band, the angle $\theta -{\theta }_{0}$ is plotted as a function of the scaled distance r/a. Here, ${\theta }_{0}$ is the angle at which the traveling band attaches to the rotating core. **c** Time evolution of the slope parameter a obtained by fitting Eq. (1) to the data (blue) and ratio a=V/Ω (green) obtained from the experiments (solid-line with circle) and simulations (dotted-line with square). Data are presented as mean value ± standard deviation (SD). Sample sizes: experiment: a, *N* = 7, 8, 10, 9, 10, 11, and 12 samples; V, *N* = 8 samples; Ω, *N* = 17, 20, 18, 17, 17, 15, and 13 samples (at $t=22.5-32.5{{\rm{h}}}$); simulation: a, *N* = 8, 8, 8, 8, 9, 11, and 8 samples; V, *N* = 66, 67, 70, 67, 61, 66, and 66 samples; Ω, *N* = 10, 11, 12, 19, 12, 12, and 12 samples (at t=22.5−32.5 h). **d** Time-lapse images of spiral pattern showing that the patterns become more tilted to the circumferential direction over time. **e** Time evolution of the traveling band speed V (light blue) and the angular speed of pattern rotation Ω (magenta), from experiments (solid-line with circle) and simulations (dotted-line with square), which are the same data used to calculate a=V/Ω in (**c**). Data are presented as mean value ± standard deviation (SD). Source data are provided as a Source Data file.

Because the bands emanate from the rotating core and propagate at an almost constant speed across the field, the resulting spiral morphology should be universal, independent of the specific mechanism that generates and transports the bands. A comparable geometry has been analyzed for spiral waves in excitable media^36,37^, where the arms follow the involute of a circle. In polar coordinates (r,θ) (Fig. 2a, Supplementary Fig. 2a), it is given by

1 $$\theta=\sqrt{{\left(r/a\right)}^{2}-1}-\arccos \left(a/r\right)-\Omega t$$

where a=V/Ω with V the speed of band propagation and Ω the angular rotation speed of the pattern (Methods). Equation (1) describes well the traveling band shapes traced at individual time points (Fig. 2b and Supplementary Fig. 1c). Over longer timescales, the parameter a, which characterizes the slope of the curve, decreased (Fig. 2c, blue circles), indicating that the spiral arms became progressively more tilted (Fig. 2d). Because the parameter a is related to V and Ω via a=V/Ω, to identify the cause of this decrease, we measured V and Ω as functions of time and found that V remained nearly constant whereas Ω increased gradually (Fig. 2e and Supplementary Fig. 2a, c, d). The slope parameter a obtained from the band shapes matched the ratio V/Ω (Fig. 2c), confirming that Eq. (1) reliably describes our system.

### Alignment interactions promote polar order

**Fig. 3:**
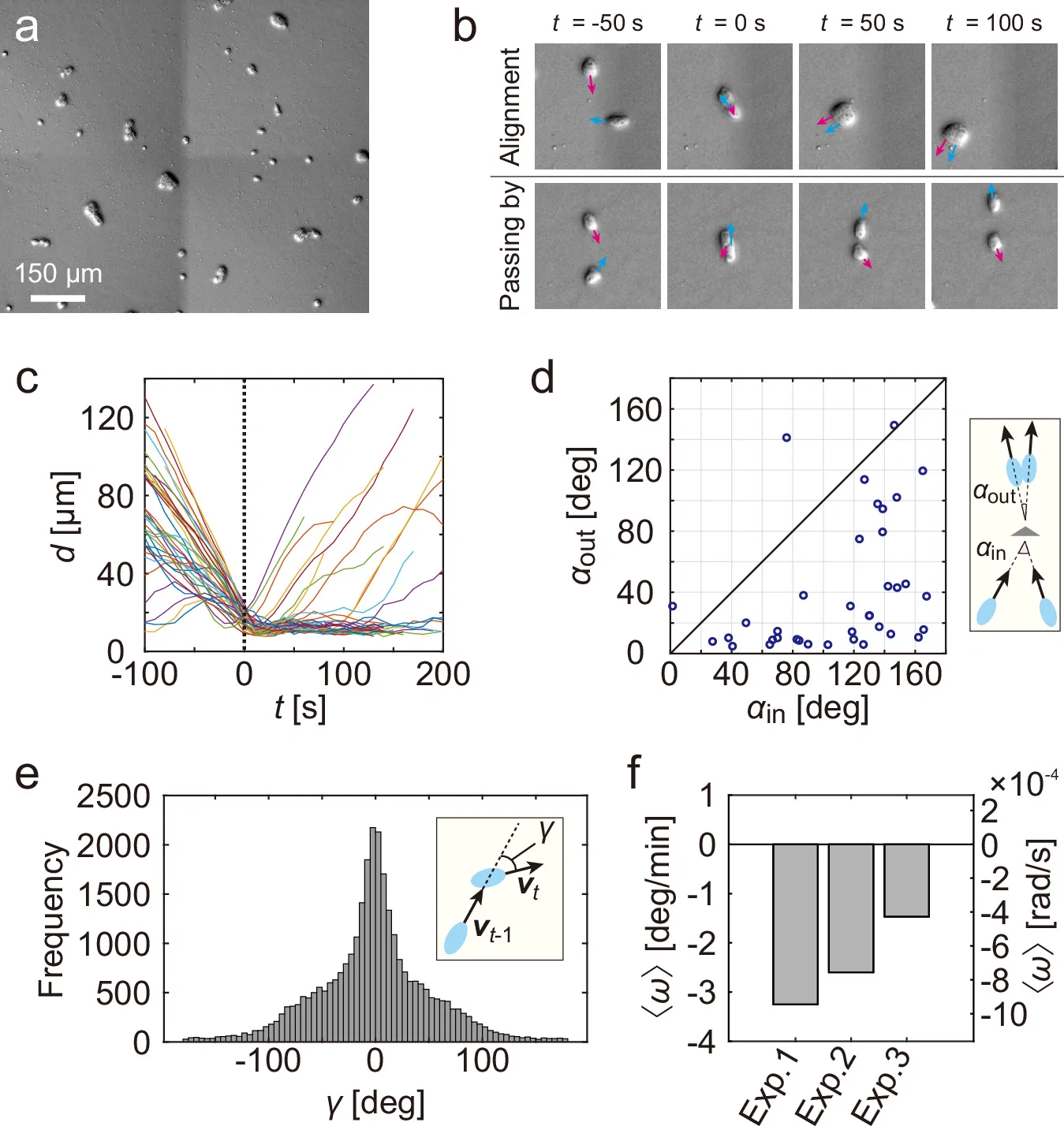
Alignment interaction and origin of chirality. **a** Dynamic cluster formation with repeated fusion and fission at low cell density (see also Supplementary Movie 2). **b** Interaction between two cells. Two cells formed a cluster and showed an alignment of migration direction (top) or passed by (bottom) upon collisions. **c** Distance between two cells before and after collision. Collision occurred at t=0. After the collisions, the distances remained close to the cell size in most cases, indicating that they often formed clusters. *N* = 37 collisions from *N* = 2 independent experiments. **d** Change in the relative angle of the migration directions upon two-cell collision. Most points are distributed below the diagonal line (y=x), indicating that the outgoing angle ${\alpha }_{{{\rm{out}}}}$ tends to be smaller than the incoming angle ${\alpha }_{{{\rm{in}}}}$. *N* = 37 collisions from *N* = 2 independent experiments. **e** Distribution of angular change γ in the migration direction over 30 s. Here, we tracked the centroids of single cells and small clusters without distinguishing between them. The histogram was constructed from 29,819 data points. **f** Mean angular velocity $\left\langle \omega \right\rangle$ of the KI cells from three independent experiments. $\left\langle \omega \right\rangle$ in each experiment was significantly below zero (95% confidence interval entirely negative). Each mean value was calculated from 29,819, 17,793, and 44,340 data points (γ), respectively. Source data are provided as a Source Data file.

Polar pattern formation is probably driven by direct cell–cell interactions. To characterize these interactions, we examined sparsely seeded KI cells that often formed dynamic clusters undergoing repeated fusion and fission (Fig. 3a, and Supplementary Movie 2). When two solitary cells collided, they tended to align their migration directions (Fig. 3b). In many cases the post-collision separation remained roughly one cell diameter, indicating the formation of two-cell clusters (Fig. 3c). Quantitative analysis showed that the incoming angle ${\alpha }_{{{\rm{in}}}}$ was usually larger than the outgoing angle ${\alpha }_{{{\rm{out}}}}$ (Fig. 3d), demonstrating a systematic reduction in relative orientation and, therefore, an effective alignment interaction. This short-range alignment interaction likely drives polar pattern formation in KI cell populations.

### Origin of chirality

We next investigated the origin of chirality. A previous study showed that wild-type *Dictyostelium* cells tend to migrate clockwise^35^. We therefore asked whether this single-cell bias underlies the large-scale chirality observed in KI cell populations. To test this idea, we transferred a small number of cells from the rotating core onto a separate non-nutrient agar plate and tracked their trajectories (Supplementary Movie 3). Because cells readily adhered to one another, we analyzed both solitary cells and small clusters together, following the centroid of each object. Cluster areas ranged from $172\,{{\rm{\mu }}}{{{\rm{m}}}}^{2}$ to $1670\,{{\rm{\mu }}}{{{\rm{m}}}}^{2}$, with a mean of $1028\,{{\rm{\mu }}}{{{\rm{m}}}}^{2}$ (Supplementary Fig. 3a), suggesting that the mean cell number in a cluster was ~3.3 assuming the radius of a cell to be $10\,{{\rm{\mu }}}{{\rm{m}}}$.

From each trajectory we calculated the angular change in migration direction, γ, over a 30 s interval and converted it to an angular velocity, $\omega=\gamma /30$ s (Fig. 3e and Supplementary Fig. 3b). Although the distribution of γ spanned both positive and negative values, the mean angular velocity $\left\langle \omega \right\rangle$ was consistently negative in three independent experiments (-3.23, -2.81, −1.60 degrees per min; Fig. 3f). These deviations from zero were statistically significant, demonstrating an intrinsic chirality in the motion of single cells and microscopic clusters. The sign of this bias matches that reported for wild-type cells^35^.

### Chiral Vicsek model in an open system

To explain the emergence of the large-scale chiral pattern, we constructed a minimal theoretical model guided by our observations. The microscale polar alignment seen in pairwise collisions, together with the macroscale polar order and traveling bands within a low-density, disordered gas of randomly migrating cells, naturally motivates a Vicsek-type description^38^ augmented with intrinsic cellular chirality^13,14^. Because the number of cells outside the source grows over time and eventually exceeds 10^6^, we adopt a minimalist approach that captures the essential features by incorporating cell chirality into a Vicsek model in which cells are continuously emitted from a source. In this open chiral Vicsek model, the time evolution of the position ${{{\boldsymbol{r}}}}_{i}$ and velocity ${{{\bf{v}}}}_{i}$ of cell i are given by

2 $${{{\bf{r}}}}_{i}\left(t+1\right)={{{\bf{r}}}}_{i}\left(t\right)+{{{\bf{v}}}}_{i}\left(t+1\right)$$

3 $${{{\bf{v}}}}_{i}\left(t+1\right)={v}_{0}{{{\mathscr{R}}}}_{\alpha }\frac{{\sum }_{j\in {R}_{i}}{{{\bf{v}}}}_{j}\left(t\right)+{N}_{i}\left(t\right)\eta {\hat{{{\boldsymbol{\xi }}}}}_{i}(t)}{\left|{\sum }_{j\in {R}_{i}}{{{\bf{v}}}}_{j}\left(t\right)+{N}_{i}\left(t\right)\eta {\hat{{{\boldsymbol{\xi }}}}}_{i}(t)\right|}$$

where ${R}_{i}$ is the local neighborhood of cell i within a radius 1 centered at ${{{\bf{r}}}}_{i}$. ${N}_{i}$ is the number of cells in ${R}_{i}$, ${v}_{0}$ is the cell speed, and ${\hat{{{\boldsymbol{\xi }}}}}_{i}(t)$ is a random unit vector with the strength ${{\rm{\eta }}}$. ${{{\mathscr{R}}}}_{\alpha }$ is the rotation operator by angle α, reflecting the chirality of individual cells with $\alpha=-{{\rm{\omega }}}$ such that ${{\rm{\omega }}}$ represents clockwise chirality. Polar alignment observed in pairwise collisions in the experiment (Fig. 3b–d) can arise from steric interactions combined with self-alignment. To construct a minimal model that isolates the essential ingredients underlying large-scale chiral pattern formation, however, we replaced these microscopic cell-cell interactions with an effective polar-alignment interaction acting within an interaction range comparable to the cell size, following the minimal modeling approach of a previous study^3^. To mimic the cell source in the experiment, at each time step, $2{{\rm{\pi }}}{R}_{0}{\kappa }$ cells are added and emitted outward from the periphery of the circular source area of radius ${R}_{0}$ with the supply rate κ per unit length. For this chiral Vicsek-model simulation, ${v}_{0}=0.5$, η=0.3 and ${R}_{0}=100$. We chose the value of η near the order–disorder transition of our system. The source perimeter functions as an effective reinsertion boundary. In the experiment, cells that return to the cell source can re-emerge from a different position along their boundary. To capture this behavior in the model, when a cell arrives at this rim from the outside, it is removed, and a new cell is instantly placed at a random location on the rim, oriented to move outward. No other boundaries are imposed in the system.

**Fig. 4:**
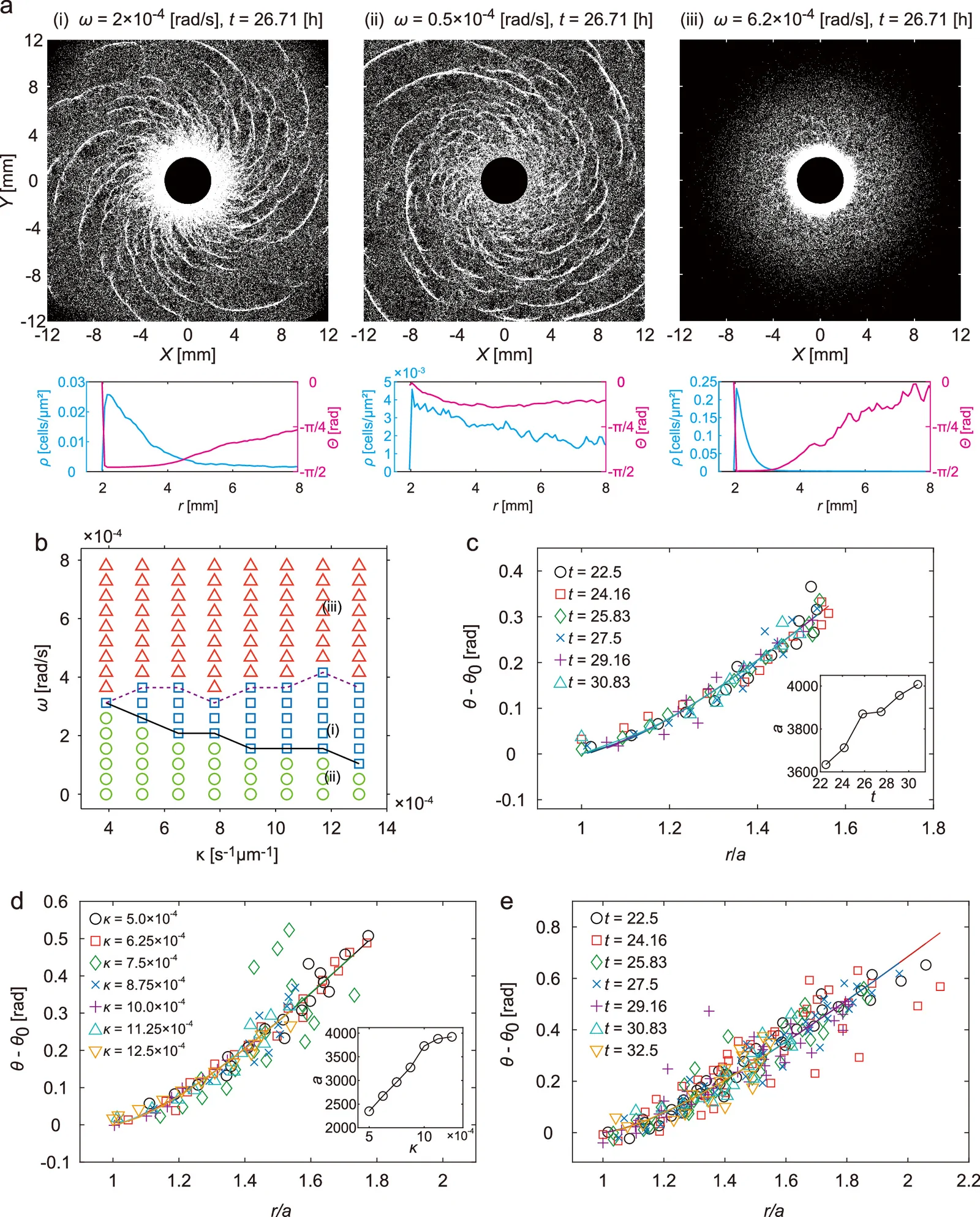
Multi-armed spiral pattern formation in an open chiral Vicsek model. **a** Three types of pattern dynamics depending on the strength of chirality ω for κ=0.45 ($1.17\times {10}^{-3}\,{{{\rm{s}}}}^{-1}{{\rm{\mu }}}{{{\rm{m}}}}^{-1}$) at time step 5000 (26.71 h). (i) Multi-armed spiral, when the chirality strength is intermediate $(\omega=0.004\,(2\times {10}^{-4}\,{{\rm{rad}}}/{{\rm{s}}}))$. (Supplementary Movie 4). (ii) Concentric pattern, when the chirality strength is small $(\omega=0.001\,(0.5\times {10}^{-4}\,{{\rm{rad}}}/{{\rm{s}}}))$. (Supplementary Movie 5). (iii) Spot, when the chirality strength is large $(\omega=0.012\,(6.2\times {10}^{-4}\,{{\rm{rad}}}/{{\rm{s}}}))$. (Supplementary Movie 6). Bottom panels indicate the density profile $\rho \left(r\right)$ along the radial direction (blue) and cell orientation relative to the radial direction $\Theta \left(r\right)$ plotted against the distance from the center (magenta). **b** Phase diagram of three different phases (multi-armed spiral (□), concentric pattern (○) and spot (△) phases) for different chirality strengths ω and the supply rate κ. The phase boundaries were determined from the two order parameters (Methods). **c**–**e** Shape analysis of the traveling band. The shape of the traveling band is well described by an involute of a circle given in Eq. (1). For each point along the traveling band, the angle $\theta -{\theta }_{0}$ is plotted as a function of the scaled distance r/a. **c** Shape analysis of the traveling band with constant supply rate κ=0.45 ($1.17\times {10}^{-3}\,{{{\rm{s}}}}^{-1}{{\rm{\mu }}}{{{\rm{m}}}}^{-1}$). (inset) The slope parameter a plotted against time. **d** Shape analysis of the traveling band with different values of constant supply rate κ. (inset) The slope parameter a plotted against the supply rate κ. **e** Shape analysis of the traveling band with decaying supply rate κ(t) with $\kappa \left(0\right)=40$ ($0.104\,{{{\rm{s}}}}^{-1}{{\rm{\mu }}}{{{\rm{m}}}}^{-1}$), β=0.02 ($0.00104\,{{{\rm{s}}}}^{-1}$), and ω=0.005 ($2.6\times {10}^{-4}$ rad/s). (Supplementary Movie 7). Source data are provided as a Source Data file.

Our model reproduces the large-scale chiral pattern observed in the experiments. A clockwise-rotating core forms around the cell source, and multi-armed spirals extend outward from the core (Fig. 4a (i) and Supplementary Movie 4). The core is strongly polar-ordered and rotates clockwise, with the polar order parameter close to unity (Supplementary Fig. 4a). Outside the core, polar-ordered bands propagate clockwise through a low-density, disordered gas of randomly migrating cells, continuously recruiting cells at the leading edge and releasing cells at the trailing edge. The radial density profile shows a peak within the rotating core (Fig. 4a (i), bottom).

The mean cell orientation relative to the radial direction, $\Theta \left(r\right)$, plotted as a function of distance from the source, reveals a characteristic reorientation sequence. Cells initially move directly outward owing to the outward orientation imposed at the source boundary. They then bend clockwise and begin to circulate around the source, eventually becoming nearly tangentially aligned. After crossing the outer edge of the core and forming spiral arms, the cells gradually turn back toward the radial direction and finally disperse outward, far from the source.

To test whether this pattern formation depends on the boundary condition at the cell source, we also performed simulations with a fully reflecting boundary condition. We found that essentially the same pattern emerges under this alternative condition (Supplementary Fig. 4b), indicating that the formation of the rotating core and multi-armed spirals is not sensitive to the specific boundary implementation. In the following simulations, we use the reinsertion rule at the boundary of the source described above.

At low chirality ${{\rm{\omega }}}$, traveling bands emerge, move radially outward, and form concentric patterns around the cell source without generating a rotating core (Fig. 4a (ii), Supplementary Movie 5). The radial density profile shows a power-law decay as $\rho \left(r\right)\sim {r}^{-0.35}$ independent of ω (Fig. 4a (ii) bottom). When ω is high, a spot-like pattern appears in which only the clockwise rotating core persists, surrounded by a disordered gas of randomly migrating cells without spiral arms (Fig. 4a (iii) and Supplementary Movie 6). In this regime the radial density obeys an exponential decay $\rho \left(r\right)\sim \exp \left(-r/\lambda \right)$, and the characteristic core width follows $\lambda \sim {\omega }^{-1.2}$, so the spot radius decreases as ω increases (Fig. 4a (iii) bottom). These three different phases, (i) multi-armed spirals, (ii) concentric pattern, and (iii) spots, arise according to the chirality strength ω and the supply rate κ when the noise strength η permits optimal alignment (Fig. 4b). These three distinct phases can also be identified by two order parameters as described in Methods.

We note that the experimentally estimated strength of cellular chirality is not in exact quantitative agreement with the range of ω over which spirals are observed in the simulations (Figs. 3f, 4b). In particular, the higher range of experimentally estimated ω overlaps with phase (iii), where spot patterns are formed. This discrepancy may partly arise from the absence of volume-exclusion effects in the present model, which allows cells to accumulate in the rotating core and thereby suppresses spiral formation. Incorporating such effects may expand the parameter range of phase (i) and improve the quantitative agreement with the experimentally observed chirality strength.

When a multi-armed spiral formed, the traveling bands that radiated from the rotating core could be fitted by the involute of a circle in Eq. (1) just as in KI cells (Fig. 4c, Supplementary Fig. 4c). In the simulation, however, the slope a increased with time, which contrasts with the experimental result (Fig. 4c inset). To clarify this difference, we measured a at a fixed time while varying the supply rate κ (Fig. 4d). The slope a grew monotonically with κ (Fig. 4d inset, Supplementary Fig. 4d). This trend suggests that, in the experiment, the number of cells in the cell source gradually declined so the effective cell supply rate fell as time passed. Indeed, cell-free patches appeared between the rotating core and the source at later times (Supplementary Fig. 7), consistent with a reduced supply. However, the supply rate and its temporal changes could not be quantified directly from boundary-crossing events in the two-dimensional experimental images, because reliable segmentation and tracking are difficult near the source, where the cell density is extremely high and the possibility of multilayer formation cannot be excluded.

To test whether the slope a would decrease when the cell-supply rate falls with time, we introduced a time-dependent cell supply defined by ${{\rm{\kappa }}}\left(t+1\right)=\left(1-\beta \right){{\rm{\kappa }}}\left(t\right)$, where β is the decay rate. With this modification, the spiral arms still followed the involute of a circle given by Eq. (1) (Fig. 4e, Supplementary Fig. 4e and Supplementary Movie 7), yet the slope a now declined over time (Fig. 2c). Furthermore, the normal propagation speed of bands remained nearly constant, while the angular speed Ω grew steadily, in agreement with the experimental observations (Fig. 2e and Supplementary Fig. 2e). The slope a obtained from the band shapes matched the ratio V/Ω (Fig. 2c), confirming that Eq. (1) accurately describes the spiral geometry. This time-dependent supply rate also reproduced the experimental sequence in which the rotating core appears first and the multi-armed spiral develops later (Supplementary Movie 7).

To test whether the formation of multi-armed spirals depends on the specific details of the model, we performed additional simulations using a chiral Kuramoto–Vicsek model in an open setting (Methods). As shown in Supplementary Fig. 6a, this model also recapitulated the formation of chiral multi-armed spirals, indicating that the results are independent of whether the dynamics are formulated in discrete or continuous time. We next examined a model that incorporates more microscopic processes underlying effective polar alignment. Specifically, we considered chiral self-propelled particles with soft-core repulsion and self-alignment in an open setting (Methods). Although in this model the mutual-polar alignment is not considered directly, the outgoing angle is smaller than the incoming angle upon collision (Supplementary Fig. 6b), indicating the effective mutual alignment, as observed experimentally (Fig. 3d). As shown in Supplementary Fig. 6c, this model also produced a rotating core accompanied by multi-armed spirals, indicating that the results do not depend on the specific implementation of effective mutual alignment, whether it is introduced explicitly through direct alignment between cells or emerges indirectly through steric interactions and self-alignment. We also note that cell-cell adhesion is not required for the emergence of the pattern. Together, these three independent models reproduce the same qualitative behavior, demonstrating that the emergence of chiral multi-armed spirals is robust across distinct modeling frameworks and arises from the combination of intrinsic chirality, effective mutual polar alignment, and continuous cell supply from a localized source, rather than from the specific implementation of cell–cell interactions.

How does clockwise collective cell migration arise within the rotating core? In our open system, continuous cell supply generates a radial density gradient, ${\partial }_{r}\rho \left(r\right)$. Such a gradient may provide a route by which microscopic chirality is converted into a net tangential bias. To examine this possibility, we analyzed the simulation data shown in Fig. 4a and measured the mean orientation of cells relative to the radial direction at r, Θ(r) (see Methods). When ω was sufficiently large to sustain a rotating core, the data approximately followed the empirical trend $\tan \Theta \sim -\omega \sqrt{\left|{\partial }_{r}\rho \left(r\right)/\omega \right|}$ (Supplementary Fig. 8). These results may indicate that the radial density gradient generated by continuous supply contributes to converting single-cell chirality into clockwise collective flow within the rotating core.

## Discussion

Here, we show that chemotaxis-deficient *Dictyostelium discoideum* mutants (KI cells) form centimeter-scale clockwise multi-armed spirals. Previous studies reported weak single-cell chirality in *Dictyostelium* cells^35^ and, separately, polar-ordered traveling bands in migrating populations of KI cells^34^. However, these observations alone did not explain how centimeter-scale chiral structures could emerge. We find that, in an open system with continuous cell supply from a localized source, a weak microscopic chiral bias can be amplified through effective polar alignment into a polar-ordered rotating core and robust clockwise multi-armed spiral bands. Supporting this interpretation, three independent models, including a minimal open chiral Vicsek model, reproduce the key features of the experiments and demonstrate effective mutual polar alignment, intrinsic chirality, and continuous cell supply as the essential ingredients for the emergence of chiral multi-armed spirals, irrespective of the specific microscopic implementations of cellular and multicellular processes. The key advance of the present study therefore lies in reporting both a previously unrecognized macroscopic chiral pattern and the physical mechanism underlying its emergence.

In our earlier work, we reported nearly periodic traveling bands but detected no clear signs of chirality^34^. In the present study, detached bands from the rotating core propagate without noticeable rotation. Notably, in contrast to excitable media^36^, traveling bands with free ends never develop into spirals on their own. Together, these observations show that the rotating core is essential for generating the multi-armed spiral pattern in this system.

Time-lapse images show that the rotating core expands outward from the periphery of the cell source until it reaches a size that appears to be set by the supply rate. At the surface of this core an instability likely develops, creating undulations that mature into several bands moving clockwise. Because these bands travel almost directly away from the center (Fig. 1d), cells must continuously flow into them along the interface with the core to keep the bands attached, so the bands continually grow and extend outward. When the core was suppressed by lowering the amount of cell suspension loaded at the source, bands still formed but displayed no clockwise organization (Supplementary Fig. 9). These results indicate that a rotating core fed by a continuous cell supply is essential for maintaining the multi-armed spiral pattern of KI cells.

The band-propagation speed V remains constant throughout the field and over time (Figs. 1e, 2e). Because the bands move tangentially around the circular edge of the rotating core, the pattern’s angular speed Ω is set by V and the core radius. The radius gradually shrinks (Supplementary Fig. 10), most likely because the cell-supply rate declines, and this reduction in radius correspondingly raises the angular speed Ω.

The intrinsic chiral bias of cell migration is tiny (about a few degrees per minute)^35^, and is usually masked by random motility. Once the cells enter a polar-ordered state, this randomness is averaged out, allowing the bias to emerge as collective chirality. With an average cell speed of about 0.52 µm/s (Methods), the expected rotation radius is on the millimeter scale. This matches the size of the rotating core, indicating that a small single-cell chirality can be amplified into centimeter-scale collective chirality.

Using *Dictyostelium*, we demonstrated a rich interplay between chiral active matter and the continuous supply of material, leading to the emergence of large-scale chiral structures such as rotating cores and multi-armed spiral patterns. This collective chirality, observed in a cellular slime mold that retains ancestral traits reflective of early multicellular evolution, may shed light on the developmental origins of left–right asymmetry in animal body plans.

## Methods

### Cell culture and preparation for spiral formation

5LP medium (1 liter) was prepared by dissolving 5.2 g of lactose monohydrate (124-00092, FUJIFILM Wako Pure Chemical Corporation) and 5.0 g of Bacto Peptone (211677, Gibco) in 1 L of Milli-Q water, followed by autoclaving. For the preparation of the plates, 15 g of agar (Powdered Agar for Bacterial Culture Media, Shoei Kanten Co., Ltd) was added to the above before autoclaving. 1 mL of *Klebsiella aerogenes* cultured in the liquid 5LP medium overnight at 37 °C was spread on the 5LP 1.5% agar plate, and the non-chemotactic *Dictyostelium discoideum* KI-5 mutant cells were inoculated in the center of the plate. The KI-5 cells were incubated at 21 °C for approximately five days. KI-5 (NBRP ID: S00058) can be obtained from the National BioResource Project for Cellular slime molds (https://nenkin.nbrp.jp).

Following incubation, the KI cells and *Klebsiella* were harvested from the plate using phosphate buffer (PB; 3 mM ${{{\rm{Na}}}}_{2}{{{\rm{HPO}}}}_{4}\cdot 12{{{\rm{H}}}}_{2}{{\rm{O}}}$, 7 mM ${{\rm{K}}}{{{\rm{H}}}}_{2}{{\rm{P}}}{{{\rm{O}}}}_{4}$). To remove the *Klebsiella*, the suspension was centrifuged, the supernatant containing Klebsiella cells was discarded, and fresh PB was added. This process was repeated twice to obtain the highly concentrated paste-like KI-5 cell suspension. ~20 µL of the paste-like KI-5 cell suspension was loaded into a dimple that was fabricated on the surface of a non-nutrient 1.5 % agar. After a few hours, the cells began to migrate out of the pellet.

The non-nutrient gel with a dimple was prepared on the glass bottom dish (50 mm diameter, D911600, Matsunami Glass Ind., Ltd.). 9 mL of 1.5% agar solution was added, and a template of the dimple (hemispherical convex structure on a cover glass) was positioned on the surface of the solution. After the solidification, the template was gently removed. The template was fabricated by dropping a UV-curing resin onto a cover glass (24×32 mm, C024321, Matsunami Glass Ind., Ltd.), and allowing it to cure.

### Observation of the spiral pattern

All observations were made with the dish upside down to prevent fogging of the lid. Observations were conducted using an inverted microscope (TiE, Nikon, Tokyo Japan) equipped with a CMOS camera (DS-Fi3, Nikon, Tokyo, Japan). For whole-spiral observations (Fig. 1b,c and Supplementary Movie 1), the 11 × 14 tile images were taken every 10 min using a 4× phase-contrast objective. The 1 × 12 tile images of a narrow window (Fig. 1f) were taken every 15 s using a 10× DIC objective. The observation shown in Fig. 1f was performed using a different sample from that used in Fig. 1b,c.

### Image processing of the spiral pattern

Phase-contrast images of the spiral pattern were processed as follows. The process begins by applying a maximum filter (radius is 1 pixel) to the original image. This filter replaces each pixel in the image with the largest value among the neighboring pixels around it. Once the filtered image is obtained, the next step is to subtract it from the original image. Then the difference is added back to the original image. Finally, the contrast adjustment and median filtering to reduce the noise were applied. By applying this process, high cell density regions, such as the rotating core and traveling bands, become brighter, improving both visibility and ease of analysis. All processes described here were performed using ImageJ (National Institutes of Health, USA, version 1.54).

### Measurement of the band velocity

To measure the velocity of the traveling band shown in Figs. 1d, e, and 2e, first, the normal vectors perpendicular to the local tangents along the traveling band were drawn at a given point ${{\boldsymbol{p}}}$ on the band at a specific time point. In the frames before and after that, the points where the normal vectors and the traveling band intersect are defined as ${{\boldsymbol{p}}}_{-1}$ and ${{\boldsymbol{p}}}_{+1}$, respectively. Velocity ${{\boldsymbol{v}}}$ was obtained by $\frac{{{{\boldsymbol{p}}}}_{+1}-{{{\boldsymbol{p}}}}_{-1}}{2\Delta {{\rm{t}}}}$, where Δt is the time interval. For the experimental data, $\Delta t=600\,{{\rm{s}}}$, while for the simulation data, Δt=40 steps was used. In Fig. 1d, e the velocities and speeds were obtained at $t=25{{\rm{h}}}$ (sample 1), $t=26.8{{\rm{h}}}$ (sample 2) and $t=25{{\rm{h}}}$ (sample 3).

### PIV analysis

PIV analysis was performed using PIVlab^39^ implemented in MATLAB (The MathWorks, Inc. Natick, MA, version 2026a). A vector field was generated at each time step, and these were then smoothed over time using a 3-point moving average. For visualization, the color map was created without considering vector magnitude, focusing only on orientation. After smoothing, if the vector magnitude was smaller than $0.2\,{{\rm{\mu }}}{{\rm{m}}}/\min$, the point was regarded as cell-free. The global polar order parameter ϕ was obtained as $\phi=\frac{1}{N}\left|{\sum }_{i=1}^{N}\frac{{{{\boldsymbol{v}}}}_{i}}{\left|{{{\boldsymbol{v}}}}_{i}\right|}\right|$, where ${{{\boldsymbol{v}}}}_{i}$ is the velocity at grid point i obtained by PIV, and N is the number of lattices in each region.

### Spiral structure described by the involute of a circle

Here, we demonstrate that the morphology of spiral bands can be described by the involute of a circle, as given by Eq. (1)^36,37^. Consider polar coordinates (r,θ), where the shape of a spiral band is described by $\theta=f\left(r\right)-\Omega \,t$ with Ω representing the rotational speed of the spiral pattern around its center (Supplementary Fig. 2b). At time t=0, suppose the spiral band intersects the radial line extending from the rotation center at point A(r,θ). Thus, θ=f(r). After a time interval ${{\rm{d}}}t$, the band intersects the same radial line at point B, satisfying $\theta=f\left(r+{{\rm{d}}}r\right)-\Omega {{\rm{d}}}t$. Consequently, the radial displacement AB is expressed as ${{\rm{d}}}r=\Omega {{\rm{d}}}t/f^{\prime} (r)$. At $t={{\rm{d}}}t$, the spiral band intersects the circle of radius r at point C. The arc length AC along the circle is given by $r\Omega {{\rm{d}}}t$. Thus, the angle ϕ formed between the spiral band and the circle of radius r satisfies

4 $$\tan \phi=\frac{1}{{rf}^{\prime} \left(r\right)}$$

Next, the distance between two successive spiral bands at t=0 and $t={{\rm{d}}}t$ is given by $V{{\rm{d}}}t$ with V the normal propagation speed of the spiral band. Hence, we obtain

5 $$\sin \phi=\frac{V}{r\Omega }$$

Combining Eqs. (4) and (5), we derive the following differential equation

6 $${f}^{{\prime} 2}={\left(\Omega /V\right)}^{2}-1/{r}^{2}$$

Solving this differential equation yields the explicit form of $\theta=f\left(r\right)-\Omega \,t$, as presented by Eq. (1).

### Characterization of spiral arms as an involute of a circle

In each snapshot of the spiral obtained in the experiment and simulation, approximately equidistant points were placed manually on the traveling band, and the distance r from the center of the cell source ${{\rm{O}}}$ to each point was measured. The vector from ${{\rm{O}}}$ to the contact point between the rotating core and the traveling band was defined as ${r}_{{{\rm{o}}}}$. Then the angle θ between ${r}_{{{\rm{o}}}}$ and the vector to each point on the band was measured. Those measurements were performed using ImageJ (National Institutes of Health, USA, version 1.54). The optimal fitting parameters were determined using a least-squares fit using the analytical form of the involute of a circle, given in Eq. (1).

### Quantification of angular speed of pattern rotation

To obtain the angular speed of the pattern rotation Ω, we first considered the cell density along a circle centered at the cell source with the radius which is larger than the size of rotating core (Supplementary Fig. 2a). Then, the cell density along the circle is plotted with time as a kymograph (Supplementary Fig. 2c–e). The high-density regions form lines rising from left to right. The slope of this line gives the angular speed of the pattern. In the simulation, kymograph images were analyzed by first dividing them into panels corresponding to different time windows (Supplementary Fig. 2e). Each panel was converted to grayscale and thresholded to obtain a binary mask of high-density regions. The mask was refined by morphological operations, including small-object removal, local bridging by elongated closing, and dilation, to connect fragmented structures. The refined mask was skeletonized to obtain one-pixel-wide density ridges. Straight line segments were then detected by a probabilistic Hough transform and filtered by length and orientation. Nearly parallel or overlapping segments were removed using angular and distance-based criteria to avoid duplicate detections. After excluding spurious segments, the remaining segments were fitted by lines (Supplementary Fig. 2e magenta line). The slopes of the lines were then converted into the angular velocities using the temporal and angular scales of the kymograph. The spread of the slopes was used to estimate the uncertainty.

### Characterization of the alignment interaction

The cells were carefully scraped from the rotating core with a pipette tip (tip diameter ~1 mm) and transferred onto a non-nutrient agar plate. Immediately after the transfer, cells were observed as aggregates. After 2 h of incubation at 21 °C, the aggregate dispersed owing to the migratory activity of individual cells. The behavior of the cells in this state was recorded under the inverted microscope with a 10× DIC objective. 2 × 2 tiled images were acquired at 10-s intervals. Individual cell-cell collision events were cropped, and the positions of both cells were tracked manually. Note that the moment when the cell contours contact each other was defined as t=0. The incoming angle ${\alpha }_{{{\rm{in}}}}$ was defined as the angle between velocity vectors of each cell at $t=-1\,{{\rm{frame}}}\,(-10\,{{\rm{s}}})$, whereas the outgoing angle ${\alpha }_{{{\rm{out}}}}$ was defined as the mean angle between the velocities from $t=5\,{{\rm{frame}}}\,(50\,{{\rm{s}}})$ to the final observation time. Just after the collision the cells take time to deform and their velocities fluctuate substantially, thus, the definition of ${\alpha }_{{{\rm{in}}}}$ and ${\alpha }_{{{\rm{out}}}}$ is not symmetric.

### Measurement of the chirality in cell migration

The chiral angular velocity of *Dictyostelium* cell migration is small, requiring a large number of samples for statistical confirmation. Because cells tend to adhere to one another, it is difficult to observe isolated single-cell migration with sufficient frequency. We therefore measured the migration chirality of single cells and small cell clusters. A small amount of cells was scraped from the rotating core using a pipette tip and transferred onto non-nutrient agar plate. The process by which the cells dispersed was recorded using the inverted microscope equipped with the CMOS camera.

The 1 × 4 tile images were taken every 30 s using a 4x phase-contrast objective. This experiment was conducted three times. The obtained images were used for the measurement of chirality in cell migration. The trajectories of cells and cell clusters were automatically tracked by using the TrackMate plugin in ImageJ (National Institutes of Health, USA, version 1.54). The tracker was a simple LAP tracker, and the detector was a Cellpose detector with a pre-trained custom model. To obtain only smaller clusters or single cell trajectories, trajectories of clusters with an area larger than $1670\,{{\rm{\mu }}}{{{\rm{m}}}}^{2}$ were excluded. Additionally, to exclude misidentifications, trajectories satisfying the following criteria were omitted: total tracking time shorter than 10 minutes, velocity smaller than 0.39 µm/s for all 10 consecutive frames (5 minutes), inclusion of periods with no movement, or an end-to-end distance smaller than 59 µm.

From the trajectories of individual cells and small clusters obtained in this analysis, we calculated γ at each time point and performed statistical analysis on these values. The statistical analysis was based on circular statistics, and the mean and confidence intervals were calculated using the functions circ_mean and circ_confmean in the Circular Statistics Toolbox for MATLAB (The MathWorks, Inc. Natick, MA, version 2026a). We regarded γ and thus the angular speed ω as significantly biased toward negative values when the circular mean was negative and its confidence interval did not include zero.

### Scale conversion between experiments and simulations

To compare the experimental and simulation results on common physical scales, we used the following conversion. In the experiment, the typical cell speed and cell size are ${v}_{c}=0.52\,\left({{\rm{\mu }}}{{\rm{m}}}{{{\rm{s}}}}^{-1}\right)$, estimated from the data used for quantifying the cell-cell alignment, and ${d}_{c}=20\,{{\rm{\mu }}}{{\rm{m}}}$, respectively. We define the characteristic time scale of cell motility in the experiment, ${\tau }_{e}$, as the time required for cells to move one cell diameter, given by ${\tau }_{e}={d}_{c}/{v}_{c}=38.46\,({{\rm{s}}})$. In the simulation, the corresponding characteristic time, ${\tau }_{s}$, is defined as the time required for a particle to move one interaction length. Because the interaction distance is set to unity and the particle speed is ${v}_{0}=0.5$ length unit per time step, we obtain ${\tau }_{s}=1/{v}_{0}=2\,{{\rm{time\; steps}}}$. Therefore, one simulation time step corresponds to ${\tau }_{r}=19.23\,({{\rm{s}}})$ in the experiment.

Assuming that the interaction distance in the simulation is taken to correspond to the cell size, one simulation length unit corresponds to $20\,{{\rm{\mu }}}{{\rm{m}}}$. Accordingly, the speed of one length unit per time step in the simulation corresponds to ${v}_{r}=20/{\tau }_{r}=1.04\,\left({{\rm{\mu }}}{{\rm{m}}}{{{\rm{s}}}}^{-1}\right).$ Similarly, an angular velocity of one radian per time step in the simulation corresponds to ${\omega }_{r}=1/{\tau }_{r}=0.0520\,\left({{\rm{rad}}}{{{\rm{s}}}}^{-1}\right)$. Using this conversion, the simulation parameter ω=0.004 rad per time step corresponds to 0.715 (deg min^-1^), which is of the same order as the experimental observation. Likewise, ${R}_{0}=100$ corresponds to 2 mm in the experiment. We used the corresponding conversion factor for length, speed, and angular speed to plot the simulation results in Fig. 2c, e.

### Three phases characterized by order parameters

To characterize the three distinct phases shown in the main text, we introduce two pseudoscalar order parameters. First, to quantify coherent rotational motion of the cells, we define the chiral circulation order parameter ${\chi }_{v}$ defined as

7 $${\chi }_{v}=\frac{1}{N}\mathop{\sum }_{i=1}^{N}({\hat{{{\bf{r}}}}}_{i}\times {\hat{{{\bf{v}}}}}_{i}) \cdot \hat{{{\bf{z}}}}$$

where ${\hat{{{\bf{r}}}}}_{i}$ and ${\hat{{{\bf{v}}}}}_{i}$ denote the unit vectors of position vector ${{{\bf{r}}}}_{i}$ and the velocity vector ${{{\bf{v}}}}_{i}$ of cell i, respectively. For the concentric pattern, ${\chi }_{v}$ is expected to be close to zero, whereas$-{\chi }_{v}$ approaches one for perfect rotational motion about the center. For given κ, $-{\chi }_{v}$ increases from 0 as ω increases and saturates once the flow becomes chiral (Supplementary Fig. 5a). We identify the transition from the concentric pattern to the multi-armed spirals phase as the value of ω at which ${-\chi }_{v}$ changes most steeply (Supplementary Fig. 5a inset).

Second, to characterize the multi-armed spiral structure, we introduce a pseudoscalar chiral parameter. The system is divided into 80 concentric annuli of equal width ($200\, {{\rm{\mu }}}{{\rm{m}}}$), indexed by s=1,…,80. For each annulus, we define the complex order parameter

8 $${\psi }_{q,s}=\frac{1}{{N}_{s}}\mathop{\sum}_{i\in s}{e}^{{iq}{\theta }_{i}}$$

where q is a positive integer, ${N}_{s}$ is the number of cells in annulus s, and ${\theta }_{i}$ is the polar angle of cell i, measured about the origin. We then define the spiral chirality measure ${C}_{q}$ as

9 $${C}_{q}=\frac{{\sum}_{s}{Im}[{\psi }_{q,s}{\psi }_{q,s+1}^{*}]}{{\sum}_{s}\left|{\psi }_{q,s}{\psi }_{q,s+1}^{*}\right|+\varepsilon }$$

where $\varepsilon={10}^{-9}$ is a small positive constant introduced only to avoid division by zero. Here, we set q=30, for which $\left|{C}_{q}\right|$ is nearly maximal. A non-zero value of ${C}_{q}$ indicates the presence of a multi-armed spiral, while the sign of ${C}_{q}$ identifies its handedness. As ω increases, ${C}_{q}$ approaches zero, reflecting the collapse of the multi-armed spiral pattern (Supplementary Fig. 5b). The transition from the multi-armed spiral pattern to the spot phase is identified by the point of the steepest change in ${C}_{q}$ (Supplementary Fig. 5b, inset).

### Chiral Kuramoto–Vicsek model in an open system

We considered point-like self-propelled particles with intrinsic angular velocity ω that move at constant speed ${v}_{0}=0.52\,{{\rm{\mu }}}{{\rm{m}}}/{{\rm{s}}}$ subjected to rotational noise and local alignment interactions. The position ${{{\bf{r}}}}_{i}=({x}_{i},\,{y}_{i})$ and orientation ${\theta }_{i}$ of cell i evolve as

10 $$\dot{{{{\boldsymbol{r}}}}_{i}}={v}_{0}{{\boldsymbol{e}}}\left({\theta }_{i}\right)$$

11 $${\dot{\theta }}_{i}=-\omega+K{\left\langle \sin ({\theta }_{j}-{\theta }_{i})\right\rangle }_{j \sim i}+\sqrt{2{D}_{r}}{\eta }_{i}\left(t\right)$$

where ${{\boldsymbol{e}}}\left({\theta }_{i}\right)=\left(\cos {\theta }_{i},\sin {\theta }_{i}\right)$, K is the alignment strength, ${\left\langle \cdots \right\rangle }_{j \sim i}$ is the average taken over all particles j within the interaction range $20\,{{\rm{\mu }}}{{\rm{m}}}$ of particle i. Here, ω is the angular velocity in the clockwise direction. The last term represents rotational noise, where ${D}_{r}$ is the rotational diffusion coefficient, estimated as 1/320 ${{{\rm{s}}}}^{-1}$^40^, and ${\eta }_{i}\left(t\right)$ is Gaussian white noise with zero mean and $\left\langle {\eta }_{i}\left(t\right){\eta }_{j}\left(t^{\prime} \right)\right\rangle={\delta }_{{ij}}\delta \left(t-t^{\prime} \right)$.

As in the model described in the main text, particles are supplied at the boundary of a circular source with radius ${R}_{0}=2\,{{\rm{mm}}}.$ The supply rate per unit length is denoted by $\kappa \left(t\right)$, so that the number of newly added particles during one simulation time step Δt=1 (19.23 s in physical time) is given by $2\pi {R}_{0}\kappa \left(t\right)\Delta t$. The supply rate κ decays according to $\kappa={\kappa }_{0}{e}^{-\beta t}$ with initial value ${\kappa }_{0}$ and the decay rate β. A reflecting boundary condition is imposed at the source boundary. As shown in Supplementary Fig. 6a, a rotating core with multi-armed spirals is observed for the parameter values $\left(\omega,K,\,{\kappa }_{0},\,\beta \right)=\left(1.454\times {10}^{-4}{{\rm{rad}}}{{{\rm{s}}}}^{-1},1.04\times {10}^{-3}{{{\rm{s}}}}^{-1},\,0.026\,{{{\rm{s}}}}^{-1}{{{\upmu }}}{{{\rm{m}}}}^{-1},\, 3.12\,\times {10}^{-4}{{{\rm{s}}}}^{-1}\right).$

### Self-alignment-based model

The cells can achieve the observed mutual alignment (Fig. 3b–d) through steric interaction between cells with self-alignment. To see the chiral multi-armed spiral formation by such interaction, we study a model of chiral self-propelled particles that interact via soft-core repulsive interaction with self-alignment. In this model, the position ${{{\bf{r}}}}_{i}=({x}_{i},{y}_{i})$ and orientation ${\theta }_{i}$ of cell i evolve as

12 $${\dot{{{\bf{r}}}}}_{i}={v}_{0}{{\boldsymbol{e}}}\left({\theta }_{i}\right)+\mu \mathop{\sum }_{j\ne i}{{{\bf{F}}}}_{{ij}}$$

13 $${\dot{\theta }}_{i}=-\omega+K\sin \left({\psi }_{i}-{\theta }_{i}\right)+\sqrt{2{D}_{r}}{\eta }_{i}\left(t\right)$$

where μ is the mobility, ${{{\bf{F}}}}_{{ij}}$ is the steric interaction between cell i and j, and ${\psi }_{i}$ is the direction of velocity ${\dot{{{\bf{r}}}}}_{i}$. $({v}_{0},{D}_{r})=\left(0.52\frac{{{\rm{\mu }}}{{\rm{m}}}}{{{\rm{s}}}},\frac{1}{320}{{{\rm{s}}}}^{-1}\right).$ The soft-core steric interaction ${{{\bf{F}}}}_{{ij}}$ is given by

14 $${{{\bf{F}}}}_{{ij}}=\left\{\begin{array}{cc}k\left(\sigma -{r}_{{ij}}\right){\hat{{{\bf{r}}}}}_{{ij}} & \left({r}_{{ij}} < \sigma \right)\\ 0 & \left({r}_{{ij}}\ge \sigma \right)\end{array}\right.$$

where k is a constant, σ is the cell size, ${r}_{{ij}}=\left|{{{\bf{r}}}}_{i}-{{{\bf{r}}}}_{j}\right|$ and ${\hat{{{\bf{r}}}}}_{{ij}}=\left({{{\bf{r}}}}_{i}-{{{\bf{r}}}}_{j}\right)/{r}_{{ij}}$.

As in the model described in the main text, cells are supplied at the boundary of a circular source without overlap to avoid strong repulsive force between them. For this model, a constant supply rate per unit length denoted by κ was considered, so that the number of newly added particles during one simulation time step Δt=1 (19.23 s in physical time) is given by $2\pi {R}_{0}\kappa \Delta t$. As shown in Supplementary Fig. 6c, a rotating core with multi-armed spirals is observed for the parameter values $\left(\mu k,\,\omega,K,\,\kappa \right)=(0.052\,{s}^{-1},\,1.454\times {10}^{-4}{{\rm{rad}}}{{{\rm{s}}}}^{-1},\,1.04\times {10}^{-3}{{{\rm{s}}}}^{-1},\,1.3\,\times {10}^{-3}\,{{{\rm{s}}}}^{-1}{{\rm{\mu }}}{{{\rm{m}}}}^{-1}).$

### Analysis of the dependence of the polar direction on the chirality and the density gradient

The direction of polar order Θ(r) at a distance r from the center is given by $\tan \Theta ({{\rm{r}}})=\frac{{P}_{\theta }(r)}{{P}_{r}(r)}$, where ${P}_{\theta }(r)=\frac{1}{N\left(r\right){v}_{0}}{\sum }_{i}{v}_{i,\theta }(r)$ and ${P}_{r}\left(r\right)=\frac{1}{N\left(r\right){v}_{0}}{\sum }_{i}{v}_{i,r}\left(r\right).$ Here, $N\left(r\right)$ is the cell number at r, and ${v}_{i,\theta }\left(r\right)$ and ${v}_{i,r}\left(r\right)$, are the azimuthal and radial components of the velocity of cell i at r.

### Reporting summary

Further information on research design is available in the Nature Portfolio Reporting Summary linked to this article.

## Supplementary information

Supplementary Information

Description of Additional Supplementary File

Supplementary Movie 1

Supplementary Movie 2

Supplementary Movie 3

Supplementary Movie 4

Supplementary Movie 5

Supplementary Movie 6

Supplementary Movie 7

Reporting Summary

Transparent Peer Review file

## Data Availability

The image data generated and analyzed in this study, and source data for all graphs and supplement have been deposited in Zenodo (https://doi.org/10.5281/zenodo.22194603) and are also available from the corresponding author upon request. Additional data can be found in the Supplementary information and Supplementary Movies.

## Code availability

The codes used for the numerical studies have been deposited in Zenodo (https://doi.org/10.5281/zenodo.22194603) and are also available from the corresponding author upon request.
