# Supplementary material for: Emergence of chiral multi-armed spirals in an open system of migrating cells under continuous cell supply
Source: Nat Commun. 2026 Oct 26;17:10535. doi: 10.1038/s41467-026-78179-4 (PMC13710842; doi:10.1038/s41467-026-78179-4)
Supplement: Supplementary file 2 [file 41467_2026_78179_MOESM11_ESM.pdf]

nature portfolio

Peer Review File

Emergence of chiral multi-armed spirals in an open system of migrating cells under continuous cell supply

Corresponding Author: Dr Tatsuo Shibata

This file contains all reviewer reports in order by version, followed by all author rebuttals in order by version.

Version 0:

Reviewer comments:

Reviewer #1

(Remarks to the Author)
The authors demonstrate convincingly that the very weak chirality observed in the motion of individual Dicty cells (which they document in their manuscript, following a previous work from Tamada and Igarashi their ref[34]) is amplified at collective level due to effective ferromagnetic alignment between cells. Polar order emerges from this alignment, classically. However, it is not under the form of a homogeneous flocking, but rather under the form of dense traveling bands. These bands we observed before by the same authors (their ref[31]), but in experimental conditions that did not reveal obvious chirality in their motion. In this new work, the authors cleverly design an experimental setup where cells are 'emitted' from a central circular source. The traveling bands then form from the boundary of a dense central CW rotating region and grow to appear as spiral arms also rotating CW. The global chirality is thus manifest and strong.

This is a remarkable result in itself. The authors complement it by Vicsek model where particles are each endowed with the identical, weak, chirality. These Vicsek particles are 'emitted' from a central circular region inside an otherwise open space. Under fairly generic conditions, spiral traveling bands form, just like in the experiments. These bands are probably related to those long-observed in the classic Vicsek model (without chirality). But there band emerge in some intermediate global density region, whereas in the present paper this global density, in a sense, is zero. This is an interesting observation for active matter physicists in itself (even regardless to the chirality-amplifying properties of interest).

Below are remarks, suggestions, and comments that I encourage the authors to take into account.

1. the authors write "high-density traveling bands that advance through a low-density, disordered background". This is possibly misleading to non-specialists: as in the Vicsek model, the sparse cells outside the bands are moving, forming a disordered gas. Then bands recruit the gas cells they meet, and leave behind an approximately equal number of cells. The authors wording may be interpreted as some flock moving on disordered, quenched, terrain.

2. the "polar order parameter Phi" should be called global polar order parameter (measured in regions of interest)

3. rather than ref[13], or in addition to it, the authors should cite the following paper, by more or less the same authors: Phys. Rev. Lett. 133, 258302 (2024). As the author will see, it describes phenomena closer to those reported in their manuscript.

4. modeling questions: what is the value of v0 used? how important is the (rather large) value of R0 used? what happens when strict reflection is applied to cells coming back to the central disk? (rather than re-distributing them at random along its circonference)

5. I disagree with (or do not understand) the statement "For unidirectional motion to develop, chirality must couple to some spatial heterogeneity" and the reference to [36]. It seems to me that the statement in invalidated by the very modeling performed by the authors, making [36] rather irrelevant.

6. in all black and white snapshots, a density scale would be appreciated. in those coming out simulations one would appreciate some mention of the number of particles currently in the field of view. the reader might also want to be informed of when the snapshots have been taken (e.g. compared to the movies)

(Remarks on code availability)

Reviewer #2

(Remarks to the Author)
In this manuscript, the authors study chemotaxis-deficient Dictyostelium discoideum mutants (KI cells). They reveal that these cells are characterized by clockwise, multi-armed spirals, each of which is a traveling band due to effective short-range alignment forces. These spirals are guided by a polar-ordered rotating core, which determines the cell chirality. The authors develop a Vicsek-like model to confirm that intrinsic chirality is key to reproducing these findings.

Although I believe that this paper faces an interesting problem, I doubt that this manuscript contains enough novelty to justify publication in Nature Communications.

Overall, I believe that this manuscript does not add consistent insights and new results compared to Ref.34 (Tamada, A. & Igarashi, M., Nature Communications 8, 20150399 (2017)), where chirality in the same system was already observed and discussed.

In addition, I have found the section "Self-organization of multi-armed spiral structure in mutant Dictyostelium cell" hard to follow and technical.

I believe that the model introduced by the authors is quite artificial and complicated. Even if the model reproduces experiments, understanding the key elements of the model is not easy in Sec. "Chiral Vicsek model in an open system". For instance, the authors write: "When a cell arrives at this rim from the outside, it is removed, and a new cell is instantly placed at a random location on the rim, oriented to move outward." The authors should describe and explain why this choice was necessary. In other words, I suggest that the authors try to clarify their model by adding comments and explanations. In addition, could the authors justify their use of alignment interactions? Several experiments on cells have been explained through a self-alignment mechanism between polarization and velocity

In addition, could the authors clarify how they distinguish between the different phases in Fig.4B (phase diagram)? What is the order parameter considered here?

(Remarks on code availability)

Reviewer #3

(Remarks to the Author)
Hayakawa et al. investigate how weak microscopic chirality can generate robust macroscopic chiral order in open active systems containing a source where elements are continuously supplied. Using chemotaxis-deficient Dicty cells, the authors find that cells emerging from a localized source form centimeter-scale clockwise rotating multi-arm spirals, with each spiral arm corresponding to a traveling, polar-ordered density band. They quantify the velocity and shape of these arms, as well as the subtle single-cell level clockwise bias. Using an open chiral Vicsek model with an intrinsic rotational bias and continuous particle injection, they then show that these ingredients are sufficient to produce the rotating core and the spiral arms. They perform a parameter scan of the chirality strength and the supply rate, and show how the macroscopic regimes depend on these parameters.
The work proposes a general mechanism by which microscopic chirality plus sustained flux yields macroscopic chiral patterning, with implications for multicellular organization and left–right asymmetry.

The connection between the weak microscopic chirality at the cellular level and the striking collective dynamics is beautiful. The influence of the cell supply rate is particularly interesting. The experimental platform, with its good access to quantification, could provide an opportunity to investigate some of these effects more quantitatively. Especially if the quantitative links between the simulations and the data can be improved, this study could constitute one of the clearest experimental validations of chiral Vicsek-type physics in a living system. However, a major limitation in directly connecting the data with the simulations appears to be the lack of time-scale calibration (see below). If this issue could be overcome, the study would be significantly strengthened.

Specific suggestions:

Overall, it remained a bit unclear to me to which extent the simulations provided evidence that the weak chiral bias at the single cell level is sufficient to *quantitatively* explain the features of the large-scale spirals, i.e. the velocity, spacing of the arms etc. The observables on the morphology and dynamics of the arms are currently shown separately for experiments and

simulations in Figures 2 and 4, using different scales and units, making a comparison difficult. Could the simulation parameters that best correspond to the experimental observations not be identified systematically? Related points:
- It appears that time is counted in terms of simulation steps in Fig. 4 (though it's not specified in the panel or the caption). Nonetheless, the magnitude of the velocity reported in Fig. 4 seems to match the measured V in physical time in Fig. 2. Could the authors not calibrate the simulation timescale, e.g. either by treating the simulation timestep as a free parameter, or by matching it to the known average velocity of a Dicty cell or similar?
- The authors are experimentally quantifying the strength of chirality omega, in degrees per minute, and later investigate systematically in the chiral Vicsek model, how weak particle-level chirality strength omega determines the macroscopic patterns. Where is the experimental system sitting in the diagram 4B? What are the units of omega in this diagram?
- The magnitude of the average rotation speed appears to be an order of magnitude off between experiments and simulations (Fig. 2E and 4F), why is this the case?
- How were the simulation parameters eta and R_0 chosen?
- Could the authors elaborate further on why experimentally quantifying the cell supply rate and its changes over time is so challenging?

Minor:
line 45: typo, should read '…of chiral active matter'

line 48-50: minor writing issue: the neural crest example comes a bit abruptly, and it's not clear why this specific example is mentioned

line 105: typo, should read ' Eq. 1 reliably describes our system.'

line 113: minor grammatical error: should read 'Quantitative analysis showed that the incoming….'

line 146 and following: \hat{v} is not defined below Eq. 3

line 148: minor grammatical error, should read '…reflecting the chirality of an individual cell…'

(Remarks on code availability)

Version 1:

Reviewer comments:

Reviewer #2

(Remarks to the Author)
I have read the new version of the paper and the reply to the Referees. In my opinion, the reply confirms the fundamental weaknesses already present in the previous version of the paper, which I discuss below. Consequently, I am against the publication of this manuscript in Nature Communications. Overall, I think that this paper does not satisfy the publishing criteria of Nature Communications, for two reasons: lack of novelty and weakness of the model.

Lack of novelty.
In response to my comment about the lack of novelty of this manuscript compared to previous results (from the same authors), the authors write:
"Ref. [33] established weak chirality at the single-cell level in Dictyostelium, and our previous study [32] reported polar-ordered traveling bands in dense migrating populations. However, these observations alone did not explain how such ingredients become coupled to generate robust centimeter-scale chiral structures. In the revised manuscript, we now clarify that the main advance of the present study is the identification of an open-system mechanism of chirality amplification, in which continuous cell supply from a localized source allows weak microscopic chirality to be amplified, through effective polar alignment, into a polar-ordered rotating core and clockwise multi-armed spiral bands."
In virtue of this reply, it is clear that this manuscript only interprets/explains phenomena previously observed by the authors (In addition, this explanation is not so strong, see below). Consequently, the paper does not satisfy the criteria of novelty and innovation to justify a publication in Nature Communications, and it is more suitable for more specialistic journals.

Weakness of the model.
To explain the results, the authors introduce a simplified model without volume exclusion and cell-cell adhesion. They write:
"In our previous studies, we used self-alignment interactions to model KI cells and showed that they can induce effective mutual alignment [32,38], although the experimental conditions were different. However, generating mutual alignment from self-alignment requires additional position-dependent pairwise interactions, such as volume exclusion and cell–cell adhesion. Such interactions are indeed present between cells, but explicitly implementing them is computationally demanding."
As they state, the only reason for the model adopted (which is simpler but highly artificial) lies in computational difficulties. Since it is quite well-known (and obvious) that cells are characterized by volume exclusion and cell-cell adhesion, it is hard for me to accept that this study is able to clarify a physical mechanism, compared to previous results.

In addition, I believe that the authors should introduce an order parameter to distinguish between different phases rather than

using visual inspection or a qualitative classification.

(Remarks on code availability)

Reviewer #3

(Remarks to the Author)
The authors have addressed my concerns. By resolving the calibration issue of time- and lengthscales they now convincingly show quantitative agreement between theoretical and experimental results.
I recommend publication.

(Remarks on code availability)

Reviewer #4

(Remarks to the Author)
The authors have done a laudable job addressing the previous' reviewers concerns.

My only remaining criticism lies in the proper referencing of the first and original pieces of work concerning active chiral matter in living systems. The two papers that started a new interest in chiral active processes in cells were first Naganathan and Grill, eLife 2014, and second Tee and Bershadsky, Nature Cell Biology 2015. The latter one is referenced, the first one is missing. It should be referenced at the same location as reference 20. Second, the Noselli paper that identifies processive myosins to overtwist and undertwist the fruit fly is also missing, it provides the best link from molecular mechanism to cell-scale and beyond chiral movements. It should also be referenced at the same place (Lebreton and Noselli, Science 2018).

Otherwise I have no remaining concerns, the discovery of large scale chiral movement in Dicystelium is as important and it is beautiful. The quantifications are appropriate, the Viscek model is an important addition, and the match between data and modeling is compelling.

I consider this to be an important addition to the field that should be published once these last concerns described above are amended.

(Remarks on code availability)

Version 2:

Reviewer comments:

Reviewer #2

(Remarks to the Author)
The authors' second reply to my comments has convinced me of the relevance of the present paper, which goes beyond merely reinterpreting previous experimental results. I believe that the additional simulation studies further support the authors' claims and the main message of the paper.

Consequently, I recommend that the paper be accepted in its current form.

(Remarks on code availability)

Open Access This Peer Review File is licensed under a Creative Commons Attribution 4.0 International License, which permits use, sharing, adaptation, distribution and reproduction in any medium or format, as long as you give appropriate credit to the original author(s) and the source, provide a link to the Creative Commons license, and indicate if changes were made.
In cases where reviewers are anonymous, credit should be given to 'Anonymous Referee' and the source.
The images or other third party material in this Peer Review File are included in the article's Creative Commons license, unless indicated otherwise in a credit line to the material. If material is not included in the article's Creative Commons license and your intended use is not permitted by statutory regulation or exceeds the permitted use, you will need to obtain permission directly from the copyright holder.
To view a copy of this license, visit https://creativecommons.org/licenses/by/4.0/
