# Supplementary material for: Emergence of chiral multi-armed spirals in an open system of migrating cells under continuous cell supply
Source: Nat Commun. 2026 Oct 26;17:10535. doi: 10.1038/s41467-026-78179-4 (PMC13710842; doi:10.1038/s41467-026-78179-4)
Supplement: Supplementary file 3 [file 41467_2026_78179_MOESM1_ESM.pdf]

Supplementary information

Emergence of chiral multi-armed spirals in an open system of migrating cells under continuous cell supply

Masayuki Hayakawa[1,2]†, Biplab Bhattacherjee[1]†, Hidekazu Kuwayama[3], Tatsuo Shibata[1]*

[1] Laboratory for Physical Biology, RIKEN Center for Biosystems Dynamics Research; Kobe 650-0047, Japan. [2] Department of Mechanical Engineering, Kyoto Institute of Technology; Kyoto 606-8585, Japan. [3] Faculty of Life and Environmental Sciences, University of Tsukuba; Tennodai, 1-1-1, Tsukuba, Ibaraki 305–8572, Japan.

*Corresponding author. Email: tatsuo.shibata@riken.jp.

†These authors contributed equally to this work

This PDF file includes:

- Supplementary Figures 1 to 10
- Legends for Supplementary Movies 1 to 7

Additional supplementary information for this manuscript includes the following:

- Supplementary Movies 1 to 7

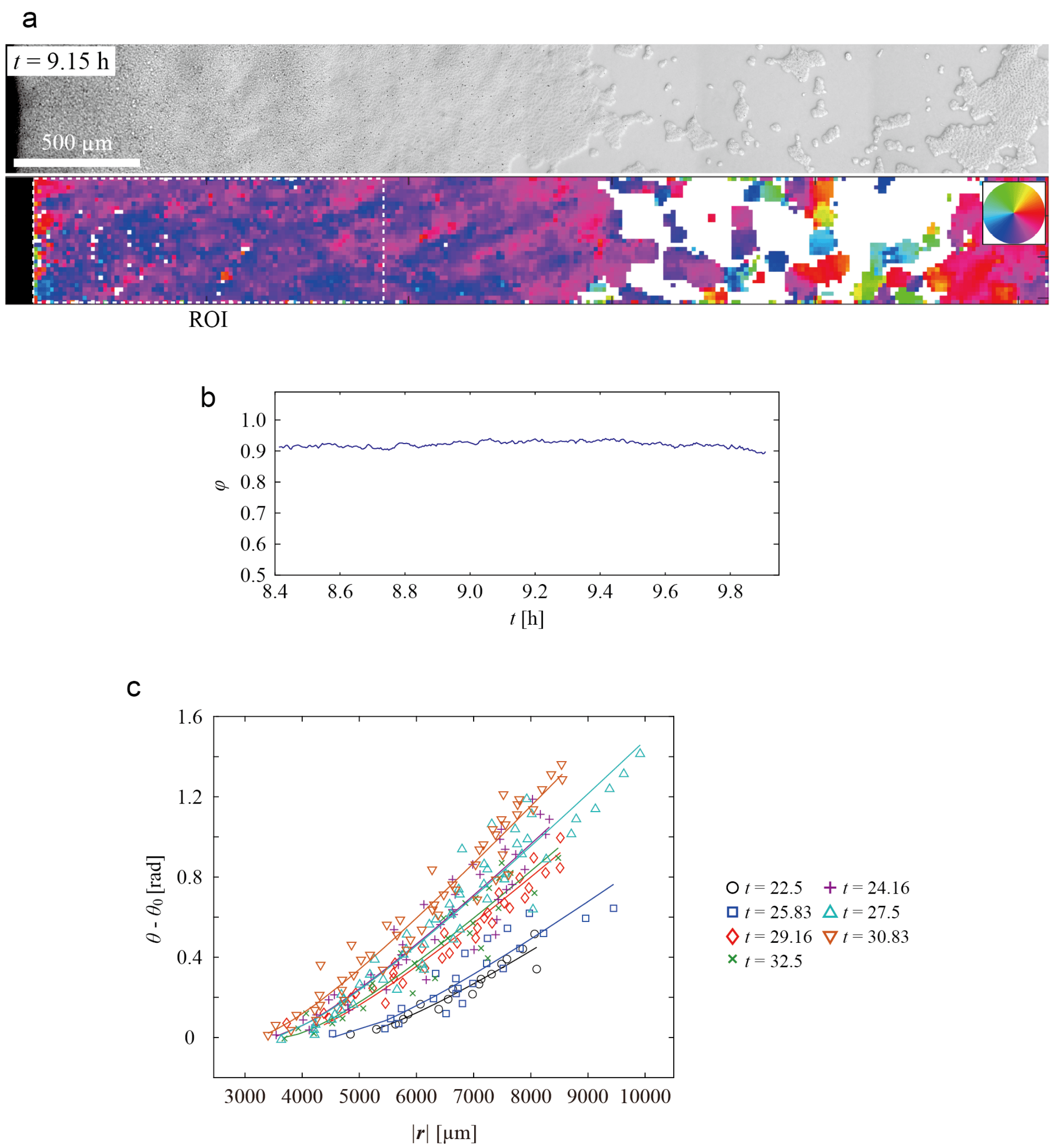

Supplementary Fig. 1. Formation of rotating core with polar order prior to the emergence of multi-armed spiral and shape of spiral arms. (**a**) A high-magnification DIC image and the particle image velocimetry (PIV) analysis along a corresponding radial window, as indicated schematically in Fig. 1C at 9.15 h. The color indicates the direction of motion obtained by PIV, as shown in the color wheel. (**b**) Time series of the global polar order parameter ϕ obtained in the region indicated in A. The high value of around $\phi \approx 0.9$ indicates that the rotating core is in a polar-ordered state. (**c**) Temporal evolution of the spiral shape shown in Fig. 2b.

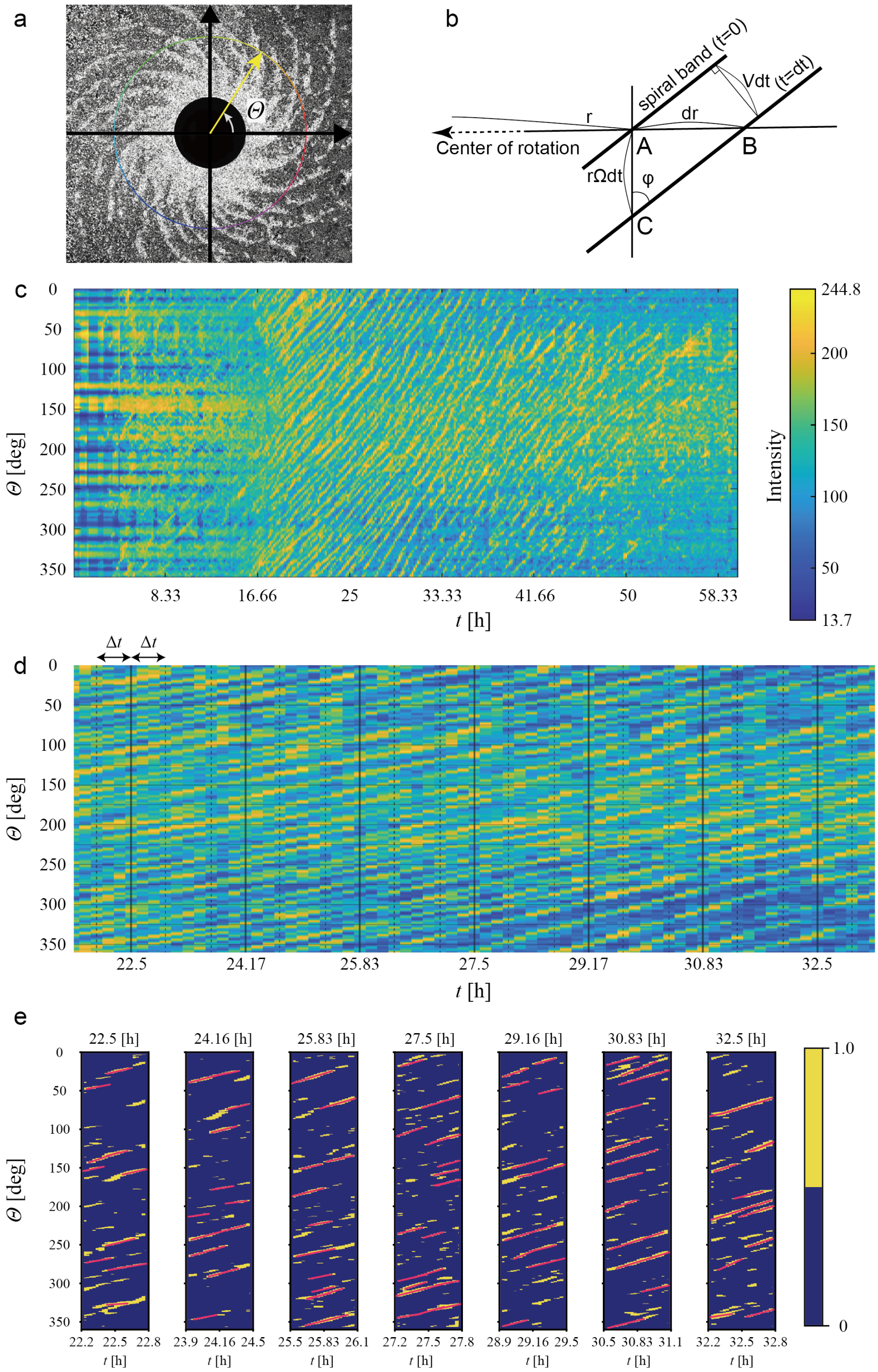

Supplementary Fig. 2. Quantification of angular speed of pattern rotation. (**a**, **c**, **d**) Experiment and (**e**) simulation of open chiral Vicsek model. (**a**) Cell density profile considered along the circle. (**b**) The spiral wave rotates about the origin through an angle Ωdt during a time interval dt, and the wavefront advances a distance Vdt along its normal. (**c**) The cell density in the angular direction Θ along the circle in (**a**) plotted over time. (**d**) The magnified image in (**c**). The slope of the high-density region gives the angular speed of pattern rotation Ω. (**e**) Cell density profile along the circle at each time point. The slope was measured within a range of ± 100 around each time point (magenta line). See Methods for the detailed description of the identification of Ω.

a

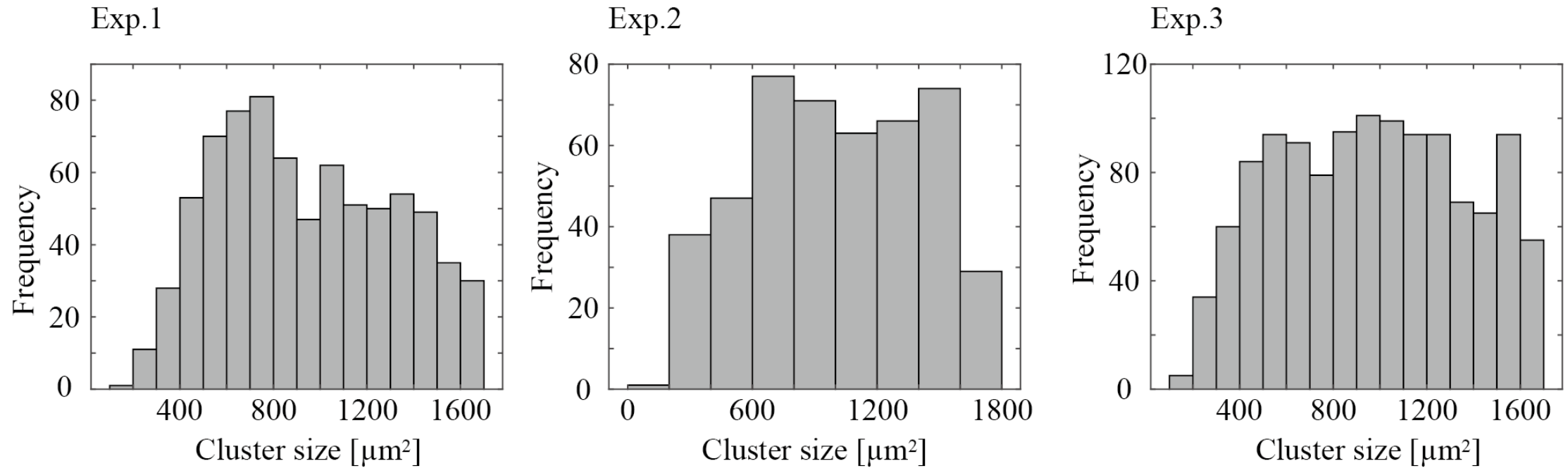

b

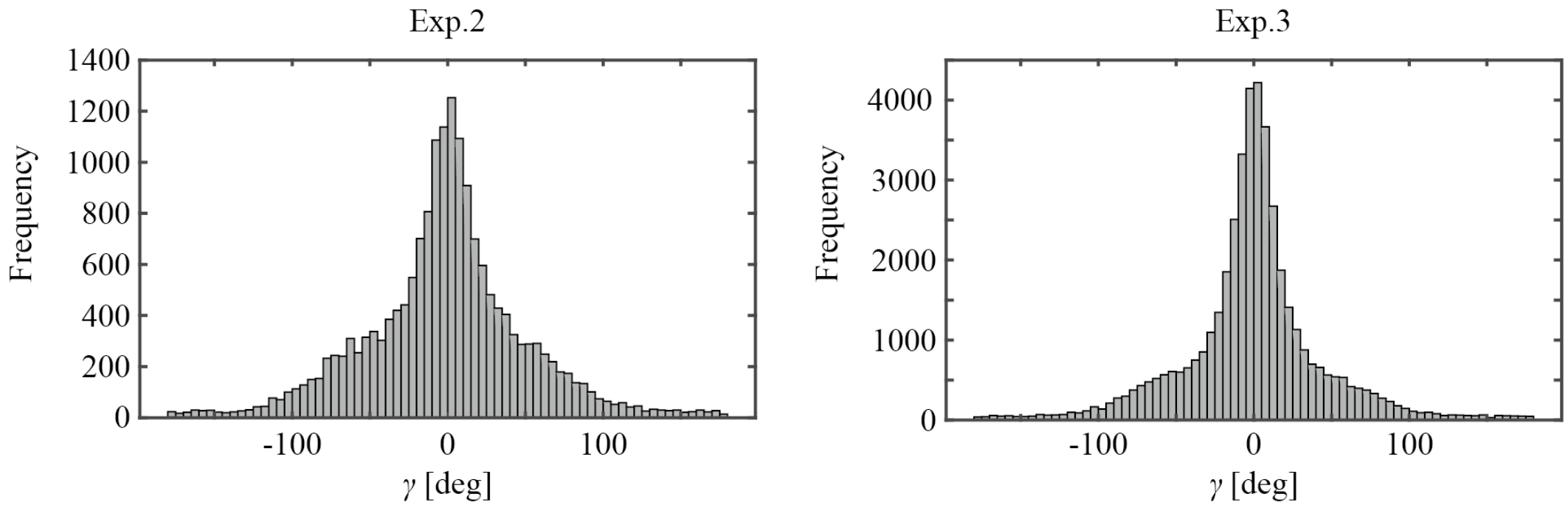

Supplementary Fig. 3. Origin of chirality. (**a**) Distribution of cell cluster area in the cell chirality assay in the three independent experiments. The size distribution of the tracked clusters ranged from $172\ \mu m^2$ to $1670\ \mu m^2$, with a mean of $1028\ \mu m^2$ for experiment 1, suggesting that the mean cell number in a cluster was approximately 3.27 assuming the radius of a cell to be $10\ \mu m$. N=763 (Exp. 1), 465 (Exp. 2), and 1211 (Exp. 3) samples from N=3 independent experiments. (**b**) Distribution of angular change γ in the cell chirality assay. Distribution of angular change in the migration direction in 30 s in the second and third experiments in addition to the first experiment shown in Fig. 3e. N=17,793 (Exp. 2), and 44,340 (Exp. 3) data points.

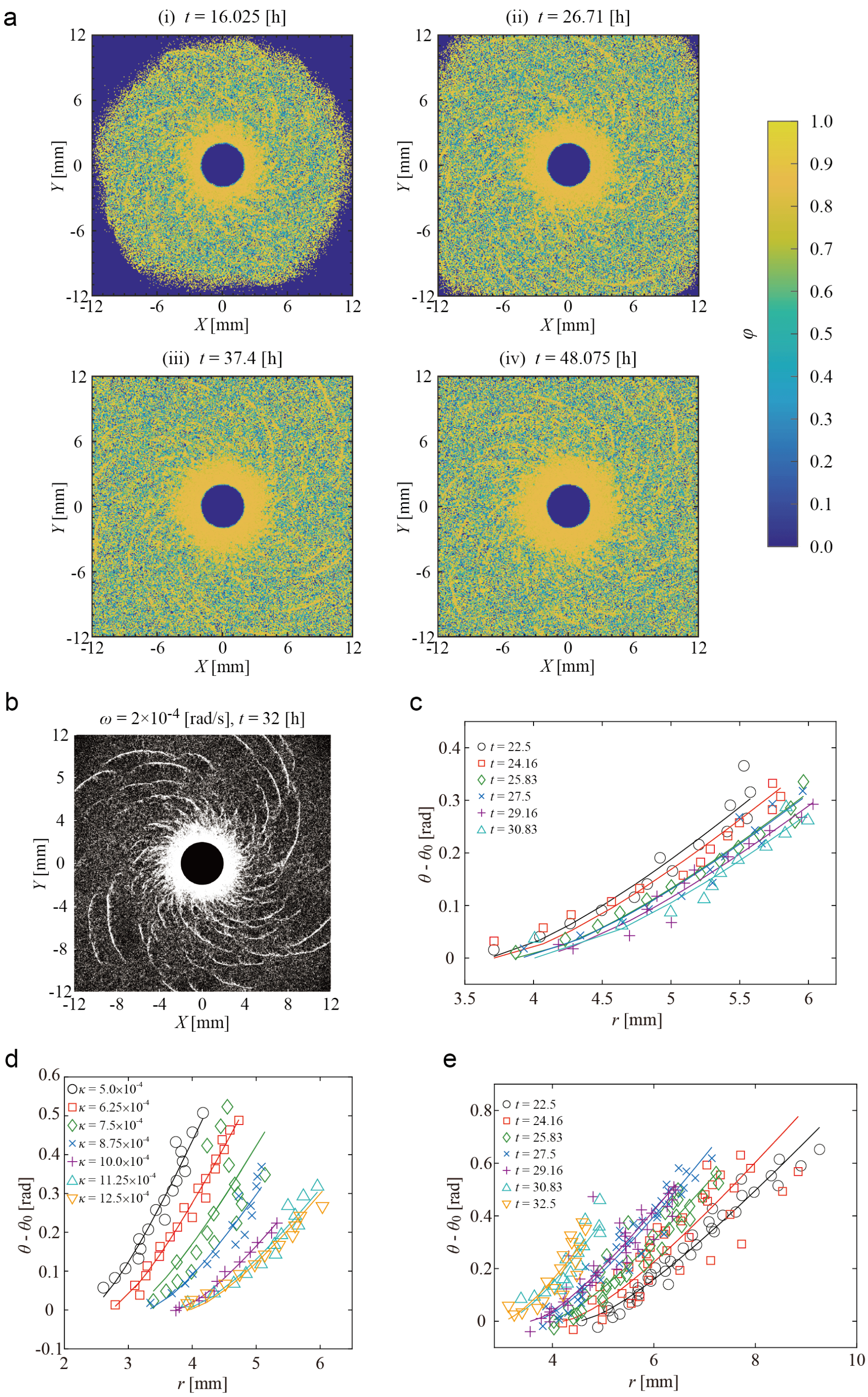

Supplementary Fig. 4. Spatiotemporal dynamics of open chiral Vicsek model. (**a**) Spatial distribution of polar order parameter measured in each local region of interest in the open chiral Vicsek model. (**b**) Snapshot from a simulation of the chiral Vicsek model in an open system with a fully reflecting boundary condition imposed at the cell-source boundary. (**c**) Temporal dynamics of the spiral shape parameterized by the slope parameter a for the case when the cell supply rate was constant shown in Fig. 4C. (**d**) Dependence of spiral shape on the supply rate κ $(s^{-1}\mu m^{-1})$ shown in Fig. 4D. (**e**) Temporal evolution of the spiral shape in the case of the time-dependent supply rate κ $(s^{-1}\mu m^{-1})$ as shown in Fig. 4e.

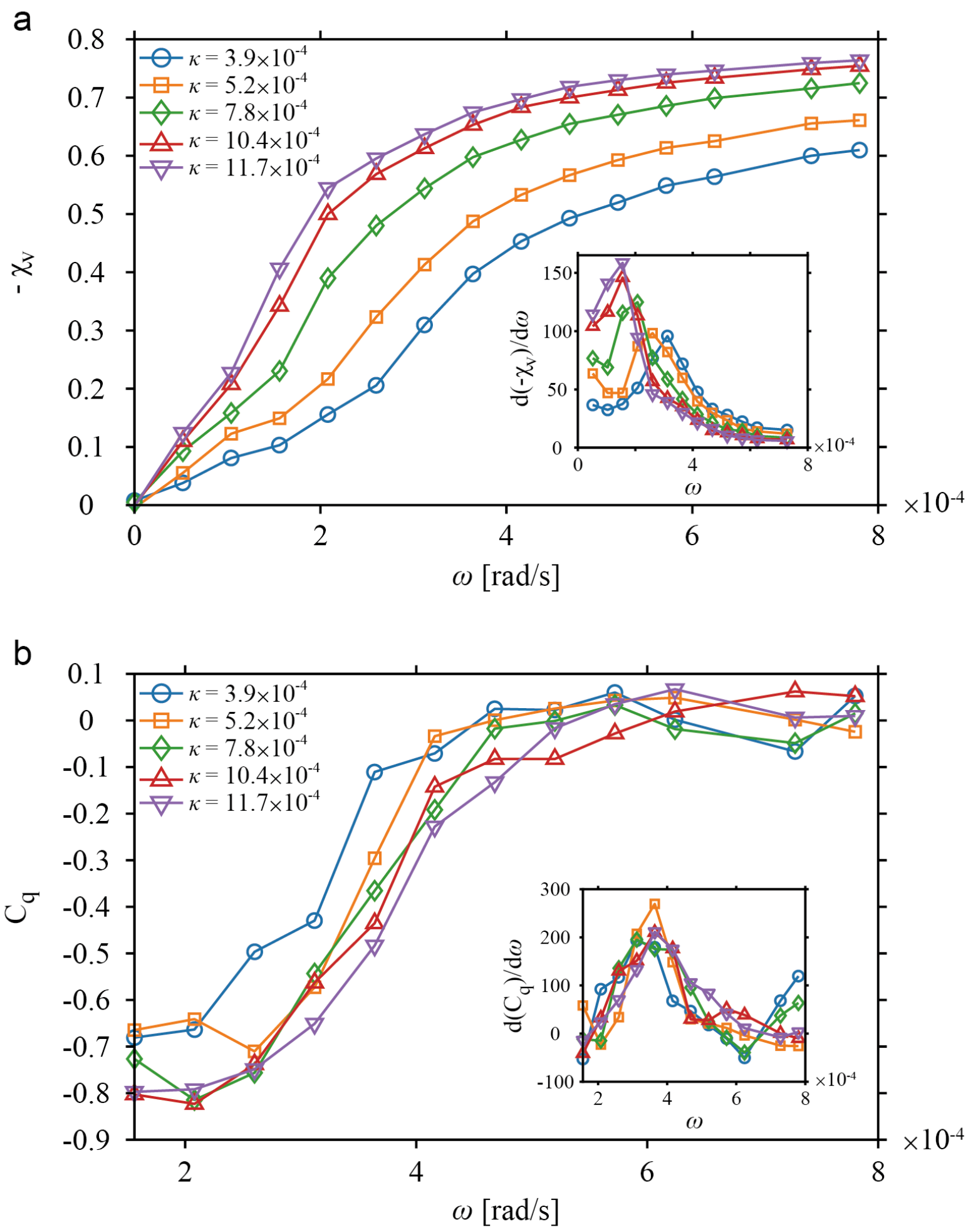

Supplementary Fig. 5. Two order parameters characterizing the three phases.

(**a**) Dependence of the negative chiral circulation $-\chi_v$ on ω for different values of the supply rate κ ($s^{-1}\mu m^{-1}$). Inset: derivative $d(-\chi_v)/d\omega$ used to identify the transition between the concentric-pattern and multi-armed-spirals phases. (**b**) Dependence of the spiral chirality measure C_q on ω for different values of the supply rate κ ($s^{-1}\mu m^{-1}$). Inset: derivative of $dC_q/d\omega$ used to identify the transition between the multi-armed-spirals and spot phases.

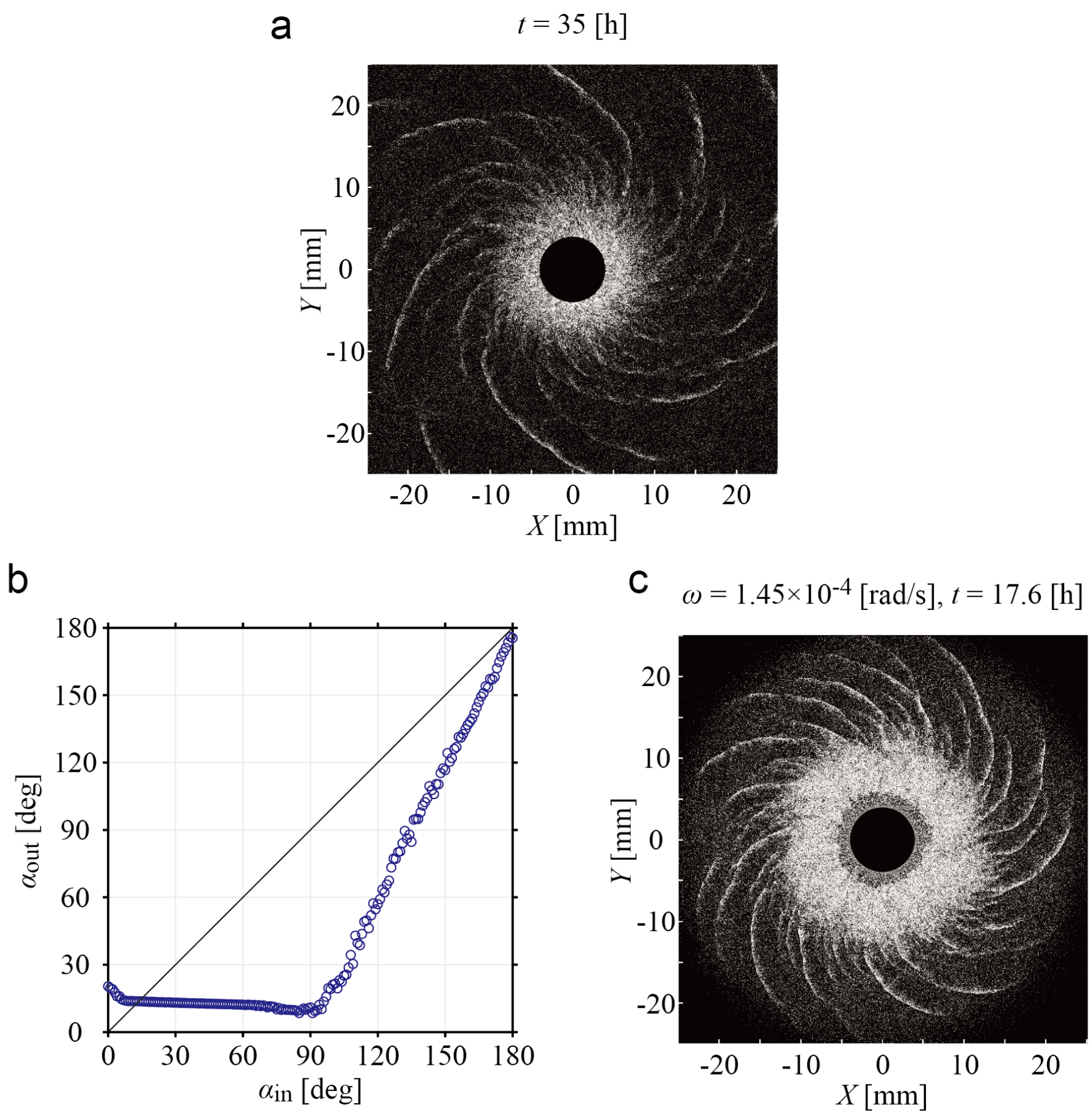

Supplementary Fig. 6. Two additional models reproducing multi-armed spirals with a rotating core. (**a**) Formation of a multi-armed spiral pattern with a rotating core in a chiral Kuramoto-Vicsek model in an open system at $t = 35$ h. See Methods for the details of the model and simulations. (**b**) Change in the relative angle of the migration directions upon two-cell collision in the self-alignment-based model, showing effective mutual polar alignment. (**c**) Formation of a multi-armed spiral pattern with a rotating core in the self-alignment-based model in an open system. See Methods for the details of the model and simulations.

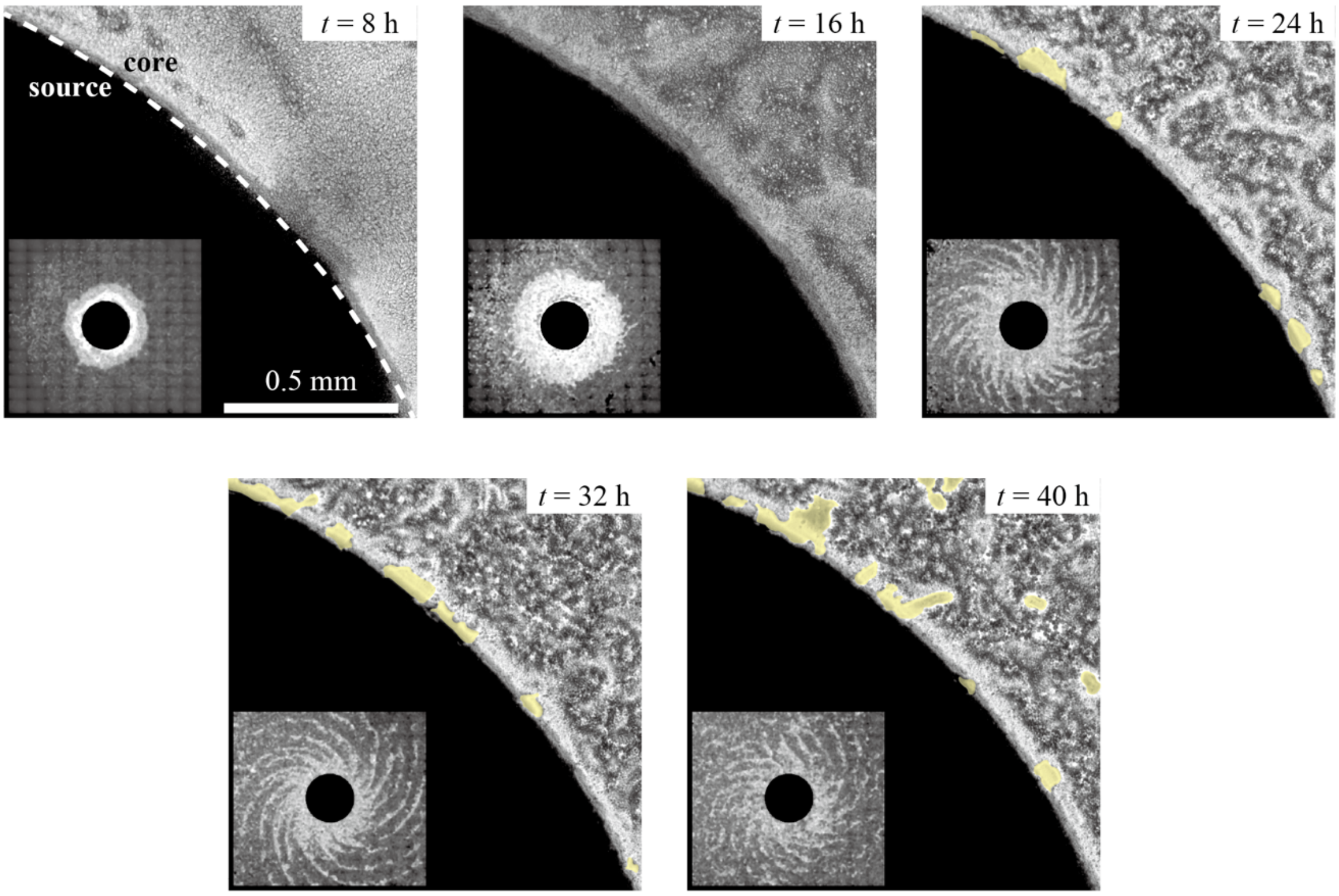

Supplementary Fig. 7. Appearance of cell-free regions near the core–source boundary. Over time, cell-free regions appear consistent with a decrease in the cell supply from the source. The yellow areas indicate the vacant spaces. The image in the bottom left of each panel shows the overall pattern at the corresponding time point.

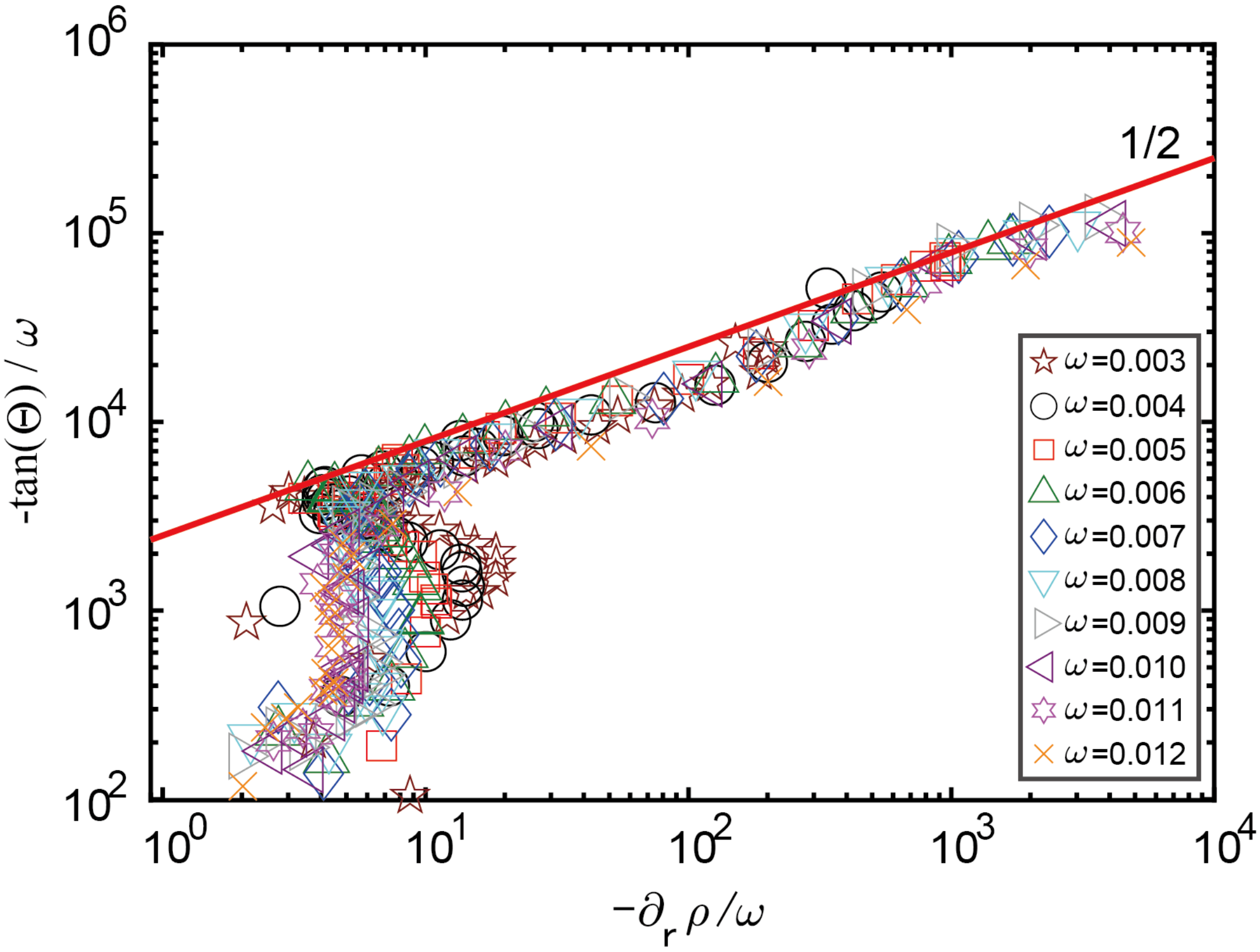

Supplementary Fig. 8. Dependence of polar order direction on the density gradient. The polar order direction $-\tan(\Theta)/\omega = -P_\theta/(P_r\omega)$ plotted as a function of the density gradient in the radial direction $-\partial_r\rho(r)/\omega$, showing a power-law dependence with exponent $1/2$, suggesting that $\tan\Theta \sim -\omega\sqrt{|\partial_r\rho(r)/\omega|}$. Here, $P_\theta = \langle v_{i,\theta}\rangle/v_0$ and $P_r = \langle v_{i,r}\rangle/v_0$ where $v_{i,\theta}$ and $v_{i,r}$ are the tangential and radial components of velocity $\mathbf{v}_i$ of cell i. Most of the points deviating from the line in the bottom-left region are from outside the rotating core.

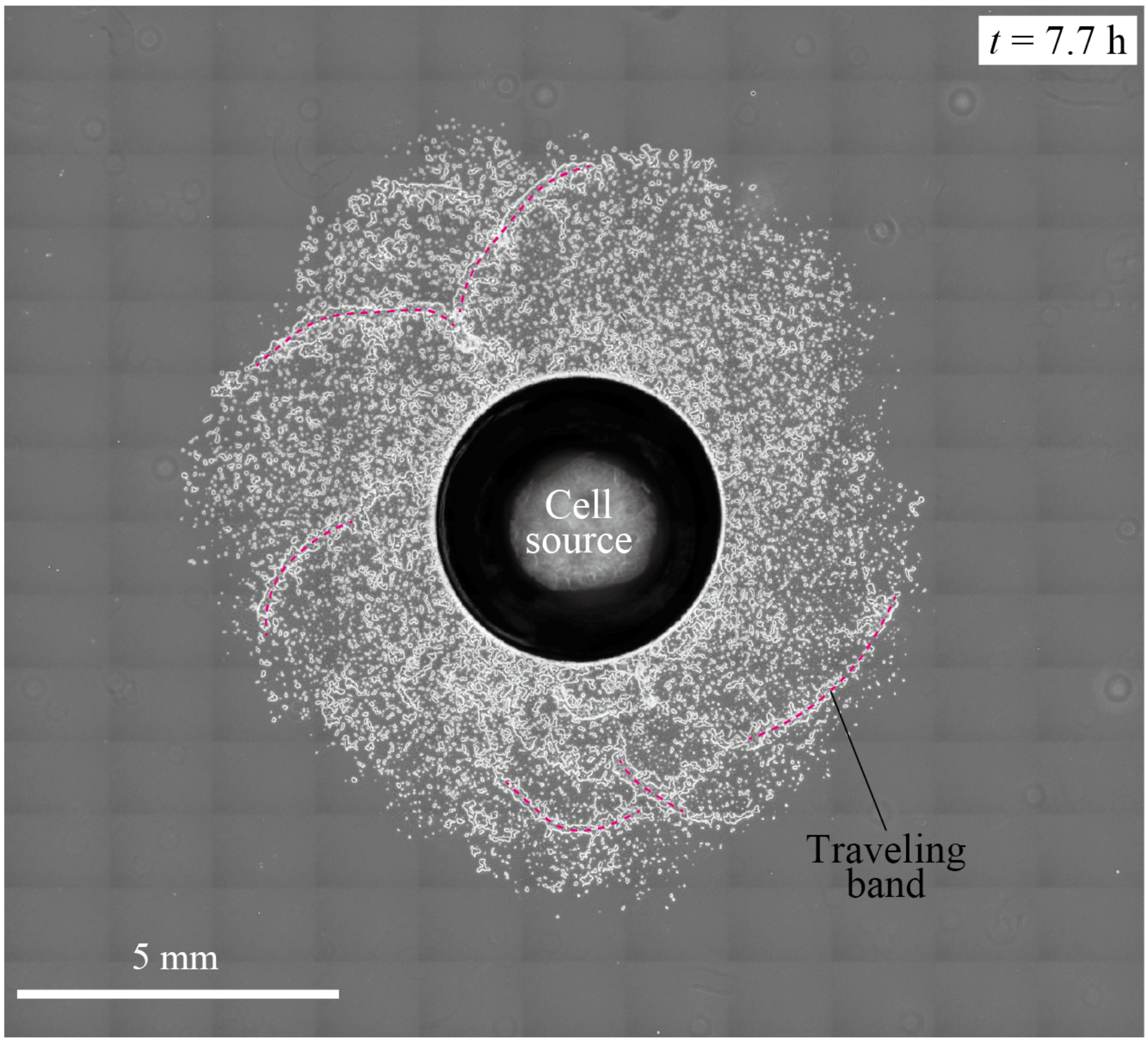

Supplementary Fig. 9. Traveling bands without rotating core.
When 3 μL of the dense cell suspension was used, traveling bands were formed without forming a rotating core. The bands propagated in random directions without forming a multi-armed spiral pattern.

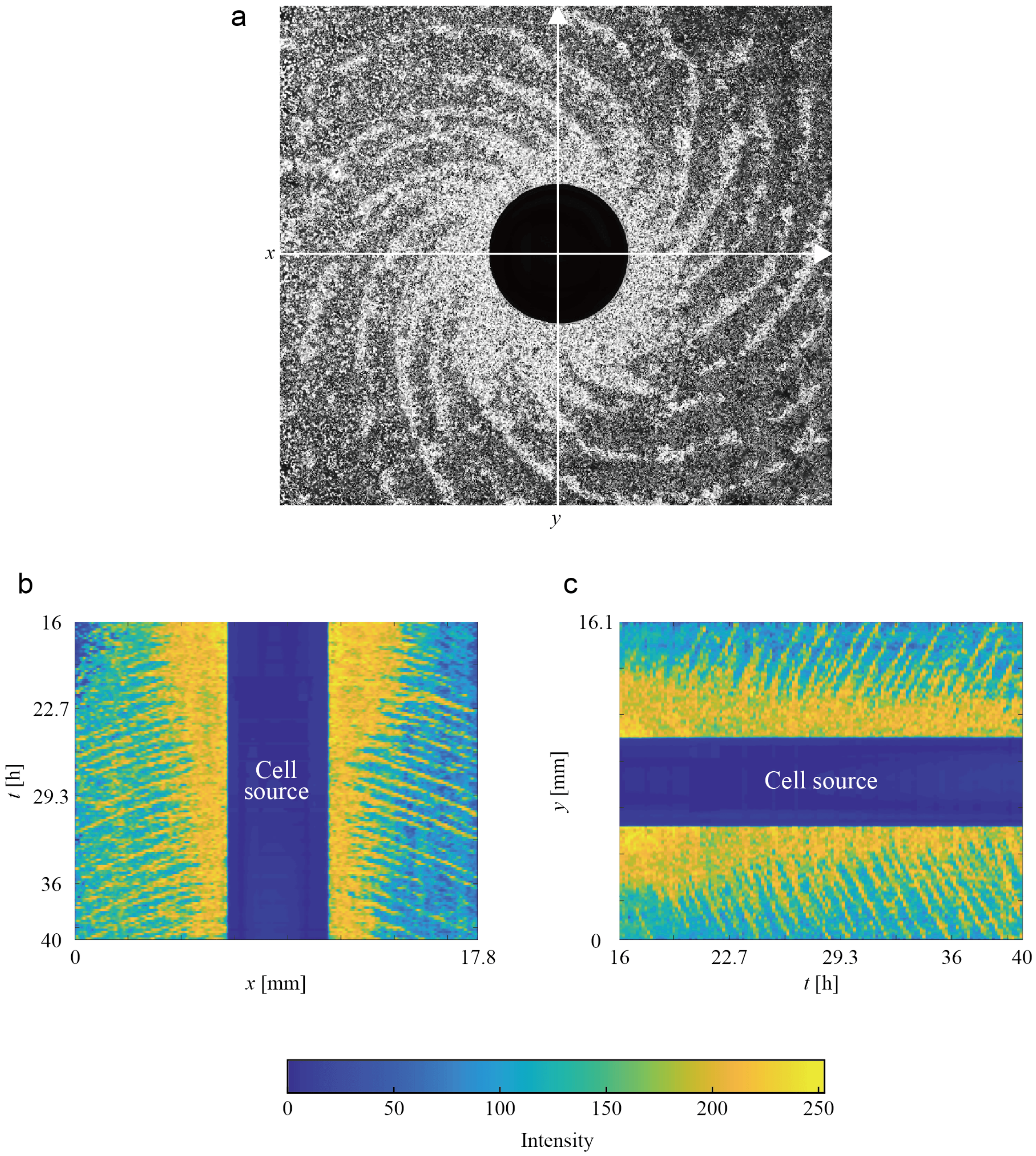

Supplementary Fig. 10. Shrinkage of the rotating core. (**a**) Cell density profile considered along the x and y axes. (**b, c**) Cell density profile along the x axis (**b**) and y axis (**c**). The width of the high cell density area around the cell source showed a decay over time.

Supplementary Movie 1. Large–scale chiral pattern formation with multi-armed spiral of KI cells.

Supplementary Movie 2. Cell-cell interaction assay at low cell density. Cells were transferred from the rotating core onto a non-nutrient agar plate. Cells frequently formed dynamic clusters undergoing repeated fusion and fission.

Supplementary Movie 3. Chirality assay of cells and cell clusters measured at low cell density. Cells were transferred from the rotating core onto a non-nutrient agar plate.

Supplementary Movie 4. Large–scale chiral pattern formation with multi-armed spirals and a rotating core in an open chiral Vicsek model shown in Fig.4A(i). $\omega = 0.004$.

Supplementary Movie 5. Concentric pattern formation in an open chiral Vicsek model shown in Fig.4A(ii). $\omega = 0.001$.

Supplementary Movie 6. Spot formation in an open chiral Vicsek model shown in Fig.4A(iii). $\omega = 0.012$.

Supplementary Movie 7. Large–scale chiral pattern formation with multi-armed spirals and a rotating core in an open chiral Vicsek model with decaying supply rate $\kappa(t)$ with $\kappa(0) = 40$, $\beta = 0.02$, and $\omega = 0.005$.
