# Supplementary material for: Emergence of chiral multi-armed spirals in an open system of migrating cells under continuous cell supply
Source: Nat Commun. 2026 Oct 26;17:10535. doi: 10.1038/s41467-026-78179-4 (PMC13710842; doi:10.1038/s41467-026-78179-4)
Supplement: Supplementary file 4 [file 41467_2026_78179_MOESM2_ESM.pdf]

Description of Additional Supplementary Files

Supplementary Movie 1. Large–scale chiral pattern formation with multi-armed spiral of KI cells.

Supplementary Movie 2. Cell-cell interaction assay at low cell density. Cells were transferred from the rotating core onto a non-nutrient agar plate. Cells frequently formed dynamic clusters undergoing repeated fusion and fission.

Supplementary Movie 3. Chirality assay of cells and cell clusters measured at low cell density. Cells were transferred from the rotating core onto a non-nutrient agar plate.

Supplementary Movie 4. Large–scale chiral pattern formation with multi-armed spirals and a rotating core in an open chiral Vicsek model shown in Fig.4A(i). ω = 0.004.

Supplementary Movie 5. Concentric pattern formation in an open chiral Vicsek model shown in Fig.4A(ii). ω = 0.001.

Supplementary Movie 6. Spot formation in an open chiral Vicsek model shown in Fig.4A(iii). ω = 0.012.

Supplementary Movie 7. Large–scale chiral pattern formation with multi-armed spirals and a rotating core in an open chiral Vicsek model with decaying supply rate $\kappa(t)$ with $\kappa(0)$ = 40, β = 0.02, and ω = 0.005.
